# Prpf4 sequentially regulates the expansion and maturation of erythrocyte through distinct mechanisms

**DOI:** 10.1038/s41420-025-02846-6

**Published:** 2025-12-08

**Authors:** Zhilin Deng, Shuying Huang, Yu Pei, Hanxiang Li, Yong Dong, Yuanyuan Li, Qin Ran, Xindong Liu, Yi Feng, Qiang Wang, Zhenhua Guo, Sizhou Huang

**Affiliations:** 1 Aging Mechanisms and Interventions Key Laboratory of Sichuan Province, Key Laboratory of Thermoregulation and Inflammation at Chengdu Medical College of Sichuan Province, School of Basic Medical Sciences, Chengdu Medical College, Chengdu, China; 2 Department of Neurology, the Second Affiliated Hospital of Chengdu Medical College, Nuclear Industry 416 Hospital, Chengdu, China; 3 Department of Cardiology, Chengdu Seventh People’s Hospital, Chengdu, China; 4 Centre for Inflammation Research, Institute for Regeneration and Repair, The University of Edinburgh, Edinburgh, UK; 5 Division of Cell, Developmental and Integrative Biology, School of Medicine, South China University of Technology, Guangzhou, Guangdong China; 6 Innovation Centre of Ministry of Education for Development and Diseases, The Sixth Affiliated Hospital, School of Medicine, South China University of Technology, Guangzhou, Guangdong China; 7 Ministry of Education Key Laboratory of Child Development and Disorders; Key Laboratory of Pediatrics in Chongqing, CSTC2009CA5002; Chongqing International Science and Technology Cooperation Center for Child Development and Disorders, Children’s Hospital of Chongqing Medical University, Chongqing, China

**Keywords:** Anaemia, Haematopoiesis

## Abstract

The proliferation of early erythrocyte and the subsequent maturation are critical events during erythropoiesis, while how these two independent but interconnected processes are efficiently orchestrated during erythropoiesis is largely unknown. *Prpf4* expression is enriched from Pre-Colony Forming Unit-Erythroid (PreCFU-E) to Nucleated Erythrocytes, especially in the CFU-E cells, implying that *Prpf4* plays a critical role in erythropoiesis. Here, we demonstrate that *prpf4* sequentially regulates erythrocyte proliferation and maturation during zebrafish definitive hematopoiesis. The data show that *prpf4* mutation results in severe defects in erythropoiesis, characterized by a substantial reduction in erythroid cell numbers and impaired erythrocyte maturation. Further analysis indicates that *prpf4* mutation leads to cell cycle arrest of erythrocytes at the S and G2/M phases, as well as a significant increase in erythrocyte apoptosis. Mechanistically, *prpf4* mutation leads to DNA damage and the subsequent activation of the DNA damage response, triggering the ATM/CHK2-p53 signaling pathway. This process inhibits the proliferation of early erythrocyte and induces erythrocyte apoptosis. On the other hand, the data reveal that *prpf4* mutation causes significant defects in skipped-exon during pre-mRNA splicing, accompanied by severe splicing defect in *slc25a39* pre-mRNA. This results in a significant downregulation of *slc25a39* mRNA, which partially impairs erythrocyte maturation during late erythropoiesis. In conclusion, we identify that *prpf4* sequentially regulates early erythrocyte proliferation and subsequent erythrocyte maturation. This dual function of *prpf4* partially explains how early erythrocyte proliferation and late maturation are efficiently coordinated during erythropoiesis.

## Introduction

Erythropoiesis is essential for maintaining normal blood function and the overall organismal health [1]. In mammals, erythropoiesis occurs in two distinct phases. The first is primordial hematopoiesis, during which primitive erythrocytes are produced in the extraembryonic yolk sac. The second wave is definitive hematopoiesis, during which all adult erythrocytes are generated in the bone marrow [2]. The process of hematopoiesis in zebrafish is remarkably similar to that in mammals and is also divided into two waves: the primordial wave and the definitive wave [3]. Specifically, the primitive erythropoiesis is confined to the posterolateral mesoderm (PLM) and gradually extends to the inner cell mass (ICM) as the embryo develops. Between 24–26 h post fertilization (hpf), with the onset of blood circulation, primitive erythrocytes enter the circulatory system. Definitive hematopoiesis begins at approximately 26 hpf, with hematopoietic stem cells (HSCs) differentiating into erythrocytes in the caudal hematopoietic tissue (CHT) around 48 hpf [4]. From 2 to 6 days post-fertilization (dpf), definitive erythroid precursors enter the bloodstream [5].

During the terminal differentiation and maturation of erythrocytes, thousands of pre-mRNAs must be accurately and efficiently spliced to accommodate extensive changes in gene expression and cellular remodeling [6]. Therefore, RNA splicing plays a critical role in erythrocyte differentiation and maturation. Previous studies have shown that CD34^+^ bone marrow cells from patients with refractory anemia with ring sideroblasts (RARS) carrying *SF3B1* mutations exhibit aberrant splicing of genes involved in hemoglobin synthesis [7]. Knocking down *SF3B1* in human CD34^+^ cells leads to apoptosis and cell cycle arrest in early erythrocytes, and also causes defective erythroblast development in later stages of erythropoiesis [8]. Further investigation confirmed that *Sf3b1* loss-of-function results in macrocytic anemia in zebrafish models [9]. In addition, mutations in the splicing factors *U2AF1* and *SRSF2* impair both hematopoietic stem and progenitor cells (HSPCs) and erythrocyte development, causing defective HSPCs development, insufficient hemoglobin synthesis and impaired erythrocyte differentiation [10, 11]. Unlike the spliceosome factors mentioned above, *ZRSR2* plays a critical role in the self-renewal of HSCs, but its function in hematopoietic differentiation and maintenance has not yet been reported [12]. These findings suggest that different splicing factors play distinct roles in erythropoiesis. However, the precise regulatory roles and mechanisms of various splicing factors in hematopoietic development remain largely unexplored.

The pre-mRNA processing factor 4 (PRPF4) is an essential component of the RNA splicing complex, playing a critical role in the assembly of U4/U6 small nuclear RNA (snRNA) and U4/U6•U5 tri-snRNPs. Previous studies have shown that overexpression of human *PRPF4*-C944T variant or knockdown of *PRPF4* disrupt photoreceptor morphology [13] and promote photoreceptor apoptosis, ultimately leading to progressive visual impairment [14]. More recently, *Prpf4* knockdown was found to impair pluripotency, proliferation, and differentiation in mouse embryonic stem cells (mESCs) [15], and *prpf4* mutations in zebrafish were shown to restrict the migration of posterior lateral line primordium (pLLP) [16]. Although the role of *prpf4* in ESCs self-renewal and pLLP migration has been elucidated, its detailed functions during embryogenesis remain largely unexplored due to the embryonic lethality in *prpf4* homozygous mutants. Gene Expression Profiling Interactive Analysis (GEPIA) revealed that *PRPF4* is highly expressed in diffuse large B-cell lymphoma (DLBC) (Fig. S1A). Further analysis of the relationship between *PRPF4* expression and survival rates in 152 patients with DLBC and AML revealed that high *PRPF4* expression was significantly associated with lower survival rates compared to patients with low PRPF4 expression (Fig. S1B). These findings suggest that *PRPF4* may play a critical role in normal hematopoiesis. In addition, analysis using BloodSpot (a database of mRNA expression of hematopoietic cells) showed that mouse *Prpf4* expression is enriched from Pre-Colony Forming Unit-Erythroid (PreCFU-E) to Nucleated Erythrocytes, with particularly high expression in CFU-E cells (Fig. S1C). This observation further implies the critical role of *Prpf4* in erythropoiesis. In this study, we demonstrated that zebrafish *prpf4* mutation results in defective erythropoiesis. Loss of *prpf4* function leads to cell cycle arrest of early erythrocytes at the S and G2/M phases, increased erythrocyte apoptosis, and impaired erythrocyte maturation. Mechanistically, *prpf4* deficiency causes the accumulation of R-loops and DNA damage in erythrocytes, which subsequently activates the ATM/Chk2-p53 signaling pathway, leading to cell cycle arrest and apoptosis. Furthermore, *prpf4* deficiency induces RNA splicing defects, resulting in significantly reduced expression of *slc25a39* mRNA and thereby blocking erythrocyte maturation. Collectively, our findings reveal that *prpf4* efficiently orchestrates erythropoiesis by regulating early erythrocyte proliferation and late-stage erythrocyte maturation.

## Results

### *prpf4* loss of function mainly causes severe defects in erythropoiesis

**Fig. 1:**
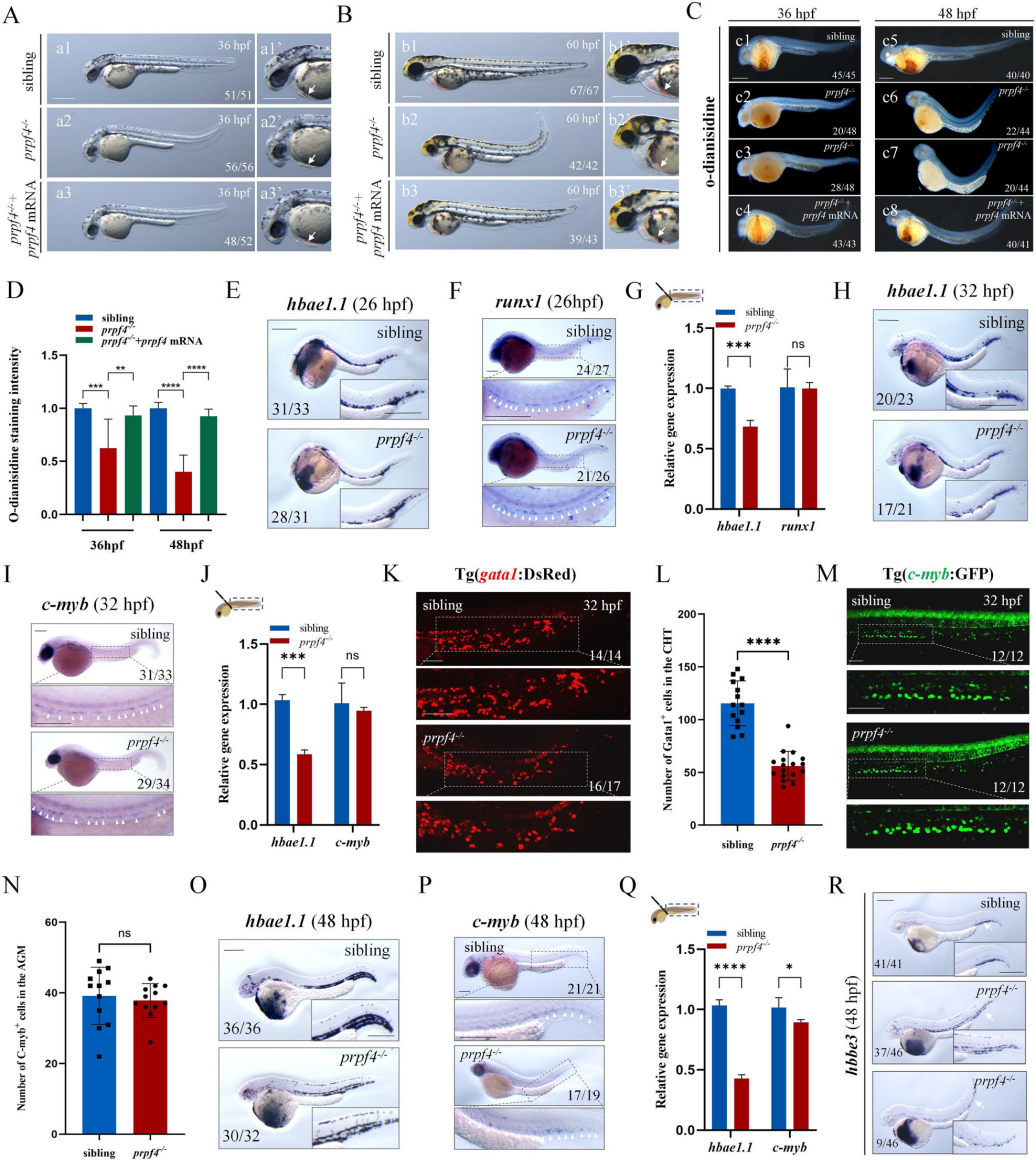
The *prpf4* mutation disrupted erythropoiesis in zebrafish. **A**, **B** Embryos with *prpf4* deficiency exhibit abnormal morphology. **A** At 36 hpf, *prpf4* mutants exhibited a slight tail curvature and a slightly paler coloration in the ventral yolk sac region (**a1–a2’**). The abnormal morphology of *prpf4* mutants was rescued by the injection of *prpf4* mRNA (**a2–a3’**). **B** At 60 hpf, *prpf4* mutants exhibited bulging ventricle, paler coloration in the cardiac region, tail curvature, and a noticeably smaller head (**b1–b2’**). The injection of *prpf4* mRNA effectively rescued the abnormal morphology of *prpf4* mutants (**b2–b3’**). The white arrows indicate the ventral yolk sac region of the embryos. **C**, **D** Detection of hemoglobin levels in siblings and *prpf4* mutants at 36 hpf and 48 hpf using o-dianisidine staining. **E–M** Analysis of erythroid and hematopoietic progenitor markers in *prpf4*^−/−^ embryos. **E** WISH showing *hbae1.1* expression in siblings and *prpf4*^−/−^ embryos at 26 hpf. **F** WISH analysis of *runx1* expression in siblings and *prpf4*^−/−^ embryos at 26 hpf. **G** RT-qPCR analysis of dissected tail regions from 26 hpf embryos. Compared with siblings (set as 1), *prpf4*^−/−^ embryos showed reduced *hbae1.1* expression (0.68-fold), while *runx1* expression remained unchanged. **H** WISH showing *hbae1.1* expression in siblings and *prpf4*^−/−^ embryos at 32 hpf. **I** WISH showing *c-myb* expression in siblings and *prpf4*^−/−^ embryos at 32 hpf. **J** RT-qPCR analysis of dissected tail regions from 32 hpf embryos. Compared with siblings (set as 1), *hbae1.1* expression was reduced in *prpf4*^−/−^ embryos (0.59-fold), whereas *c-myb* expression showed no significant difference. **K** Confocal images of Tg(*gata1*:DsRed) embryos at 32 hpf showing erythroid cells in the CHT region. **L** Quantification of Gata1⁺ cells in the CHT revealed a significant reduction in *prpf4*^−/−^ embryos compared with siblings. **M** Confocal images of Tg(*c-myb*:GFP) embryos at 32 hpf showing HSPCs in the AGM region. **N** Quantification of C-myb⁺ HSPCs in the AGM showed no significant difference between *prpf4*^−/−^ embryos and siblings. **O** WISH showing *hbae1.1* expression in siblings and *prpf4*^−/−^ embryos at 48 hpf. **P** WISH showing *c-myb* expression in siblings and *prpf4*^−/−^ embryos at 48 hpf. **Q** RT-qPCR analysis of dissected tail regions from 48 hpf embryos. *prpf4*^−/−^ embryos exhibited decreased *hbae1.1* expression (0.43-fold compared with siblings set as 1), while *c-myb* expression was reduced to 0.89-fold relative to siblings. **R** WISH showing *hbbe3* expression patterns in *prpf4* mutants and siblings at 48 hpf. Scale bars: 200 μm unless otherwise indicated; 100 μm in (**K**, **M**).

During early zebrafish development, *prpf4* is maternally expressed and widely distributed in early embryos (Fig. S2A, [16]), suggesting a potential role in regulating hematopoiesis. To examine this hypothesis, a *Tol2* transposon-mediated gene trap mutant line *prpf4*^*t243*^ was used [16]. In this mutant, The GFP signal reflects endogenous *prpf4* expression (Fig. S2B), and the fluorescence intensity allows early-stage genotype discrimination (Fig. S2C). At 36 hpf, we observed that *prpf4*^*−/−*^ embryos exhibited a slightly smaller head and a curved tail compared to controls (Fig. 1A, B), consistent with previous findings [16]. In addition, the blood in the heart region of *prpf4*^*−/−*^ embryos appeared paler compared with controls (Fig. 1Aa1’, Aa2’). At 60 hpf, *prpf4*^*−/−*^ embryos displayed more pronounced phenotypes, including a noticeably smaller head and eyes, curved tail, pericardial edema, and enlarged yolk sac, along with a marked reduction in red coloration of blood in the heart region (Fig. 1B, b1’, B, b2’). These observations suggest that *prpf4* deficiency may impair erythropoiesis in zebrafish. To confirm this hypothesis, we performed o-dianisidine staining to assess hemoglobin in erythrocytes [17]. At 36 hpf and 48 hpf, *prpf4*^*−/−*^ embryos exhibited significantly weaker o-dianisidine staining compared to controls (Fig. 1C, c1-c3, c5-c7, D). In addition, injection of *prpf4* mRNA could rescue the erythropoietic defects in *prpf4*^*−/−*^ embryos (Fig. 1A, a3, Bb3; Fig. 1C, c4, Cc8, D). These data demonstrate that *prpf4* plays a crucial role in regulating erythropoiesis.

To further investigate the role of *prpf4* in erythropoiesis, we examined the expression of hematopoiesis-related genes in *prpf4*^*−/−*^ embryos and controls at different stages. From 18 hpf to 24 hpf, no reduction in the expression of *scl*, *runx1*, *c-myb*, *gata1a*, *hbbe3*, *fli1a, hbae1.1, pu.1* or *lyz* was observed in *prpf4*^*−/−*^ embryos compared to controls (Fig. S3A–Q). This result suggests that the primary hematopoiesis was not affected in *prpf4* mutants. At 26 hpf, the beginning of the definitive hematopoiesis stage, the expression of *scl*, *gata1a*, *hbbe3*, *fli1a*, *pu.1* and *runx1* was intact in *prpf4*^*−/−*^ embryos (Fig. S4A–E and Fig. 1F, G), but the expression of *hbae1.1* was decreased (Fig. 1E, G). At 32 hpf, *hbae1.1* expression was decreased in *prpf4*^*−/−*^ embryos (Fig. 1H, J), but the HSPC marker *c-myb* expression was intact (Fig. 1I, J). Besides, we also found the number of erythrocytes decreased in *prpf4*^*−/−*^ embryos at 32 hpf (Fig. 1K,L), but the number of HSPCs was normal in *prpf4*^*−/−*^ embryos compared to controls (Fig. 1M, N). These data suggest that *prpf4* plays a critical role in erythropoiesis during definitive hematopoiesis.

Finally, we continuously examined the developmental status of erythrocytes, HSPCs, and neutrophils during later stages of definitive hematopoiesis. The neutrophil marker *lyz* and the number of neutrophils in the Tg(*lyz*:DsRed) transgenic line remained unaffected in *prpf4*^*−/−*^ embryos (Fig. S5A–C). The expression of the erythrocyte marker *hbae1.1* in the CHT was also largely reduced in *prpf4*^*−/−*^ embryos (Fig. 1O, Q; reduced to 0.43-fold of control levels). In addition, the expression of another erythrocyte marker *hbbe3* was also reduced in the CHT region of a subset of *prpf4*^−/−^ embryos (Fig. 1R). Interestingly, from 48 hpf, the expression of *c-myb* and the number of HSPCs were mildly decreased (Fig. 1P, Q; reduced to 0.89-fold of control levels; Fig. S5D, E). These data imply that *prpf4* mutation also mildly affects HSPCs development during later definitive hematopoiesis, although it is not as severe as in erythropoiesis.

### *prpf4* mutation causes erythrocyte cell cycle arrest and increased apoptosis

**Fig. 2:**
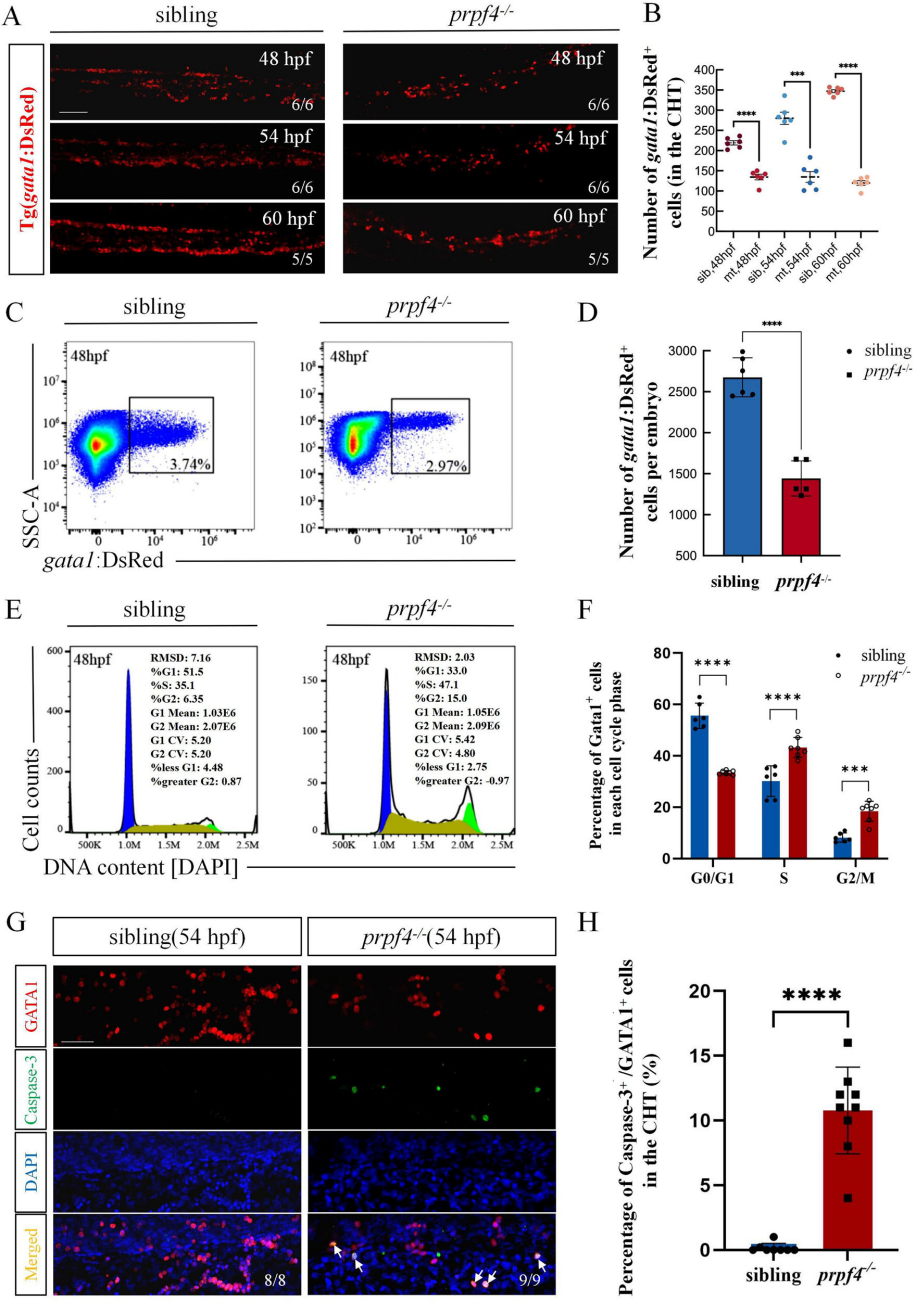
*prpf4* deficiency reduces the number of erythrocytes in zebrafish. **A**, **B** The number of erythrocytes in the CHT region of siblings and *prpf4*^−/−^ embryos was assessed using confocal microscopy in Tg(*gata1*:DsRed) transgenic zebrafish. Scale bar: 100 µm. **C**, **D** FACS analysis of GATA1^+^ erythrocytes from siblings and *prpf4*^−/−^ mutants at 48 hpf. **C** In *prpf4* mutants, the proportion of GATA1^+^ erythrocytes was reduced (30 embryos per group). **D** Quantitative analysis of GATA1^+^ erythrocyte numbers in individual embryos. **E** Cell cycle analysis of GATA1^+^ erythrocytes from siblings and *prpf4*^−/−^ mutants at 48 hpf (30 embryos per group). **F** Quantification of the percentage of cells in each cell cycle phase. **G**, **H** Confocal microscopy analysis of apoptosis in GATA1^+^ erythrocytes in the CHT region of siblings and *prpf4*^−/−^ embryos at 54 hpf. **G** Immunofluorescence staining showing GATA1 (red), active Caspase-3 (green), and DAPI (blue). Arrows indicate apoptotic GATA1^+^ cells. Scale bar: 50 µm. **H** Quantitative analysis shows a significantly higher proportion of apoptotic GATA1^+^ cells in the CHT region of *prpf4*^−/−^ embryos compared with siblings.

Since our data show that *prpf4* mutation only results in erythrocyte defects at 26 hpf and 32 hpf (Fig. 1E–N), and in the later stage (48 hpf) the erythrocyte defects were stronger than those in HSPCs (Fig. 1O–Q), we focused on investigating its function in erythroid development. First, we assessed the number of erythrocytes in the CHT region using Tg(*gata1*:DsRed) transgenic embryos at three developmental time points (48 hpf, 54 hpf, and 60 hpf). The results show that *prpf4*^*−/−*^ embryos exhibited a significant reduction in the number of erythrocytes in the CHT at 48 hpf, with a more pronounced decrease observed at 54 hpf and 60 hpf (Fig. 2A, B; Fig. S6A). Next, we performed flow cytometry analysis to quantify erythrocytes in Tg(*gata1*:DsRed) siblings and *prpf4*^*−/−*^ embryos at 48 hpf. The results demonstrate that both the ratio and number of erythrocytes were significantly reduced in *prpf4*^*−/−*^ embryos compared to siblings (Fig. 2C, D).

The significant reduction in the number of erythrocyte may result from decreased proliferation or increased apoptosis in early erythrocytes due to *prpf4* deficiency. To investigate this, we quantified the cell cycle status of erythrocytes in siblings and *prpf4*^*−/−*^ embryos by labeling DNA content with DAPI followed by flow cytometry analysis. The results show that, compared to controls, erythrocytes in *prpf4*^*−/−*^ embryos exhibited a significantly reduced proportion of cells in the G0/G1 phase, while the proportions of cells in the S and G2/M phases were significantly increased (Fig. 2E, F). These findings suggest that *prpf4* deficiency leads to cell cycle arrest of early erythrocytes in the S and G2/M phases, thereby reducing the number of cells entering the G1 phase and ultimately inhibiting overall proliferation. Next, we examined apoptosis in erythrocytes. Caspase-3 immunostaining and TUNEL assays revealed an increased number of apoptotic erythrocytes in the CHT region of *prpf4*^*−/−*^ embryos (Fig. 2G, H; Fig. S6B,C). These results indicate that *prpf4* mutation not only impairs erythrocyte proliferation but also promotes erythrocyte apoptosis during erythropoiesis.

### Upregulation of ATM/Chk2-p53 Pathway Contributes to Defective Erythropoiesis in *prpf4* Mutants

**Fig. 3:**
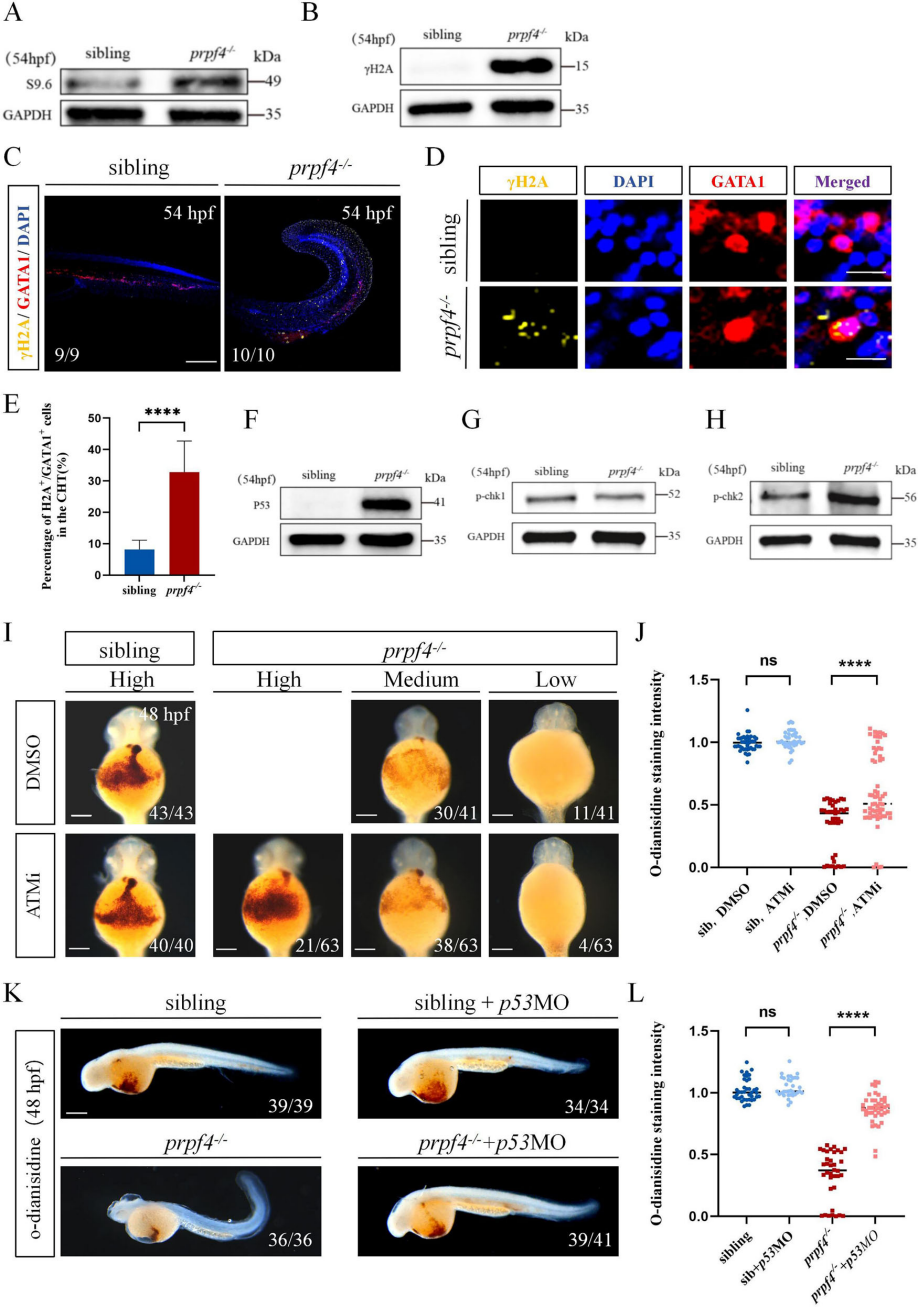
Activating of the DDR-ATM/Chk2-p53 signaling pathway disrupts erythropoiesis in *prpf4* mutants. **A**, **B** Western blot analysis for RNA:DNA hybrid accumulation and DNA damage in 54 hpf embryos. **A** S9.6 antibody detected higher RNA:DNA hybrid levels in *prpf4*^−/−^ embryos than in siblings. **B** γH2AX levels are significantly increased in *prpf4*^−/−^ embryos compared with siblings. **C–E** Analysis of DNA damage in GATA1^+^ erythrocytes in 54 hpf embryos. **C** Confocal images show γH2AX staining in the CHT region of siblings and *prpf4*^−/−^ embryos. Scale bar: 200 µm. **D** High-magnification images of erythrocytes show the colocalization of γH2AX and GATA1. Scale bar: 50 µm. **E** Quantification of the percentage of γH2AX-positive cells among GATA1^+^ cells in the CHT region. **F-H** Western blot analysis of the activation of P53 and checkpoint kinase signaling in 54 hpf embryos. **F** Increased P53 protein levels are observed in *prpf4*^−/−^ embryos compared with siblings. **G** p-Chk1 protein levels show no significant changes between *prpf4*^−/−^ embryos and siblings. **H** p-Chk2 protein levels are increased in *prpf4*^−/−^ embryos compared with siblings. **I**, **J** Effect of the specific kinase inhibitor ATMi (KU60019) on erythrocyte hemoglobin levels in *prpf4*^−/−^ embryos at 48 hpf. **I** O-dianisidine staining intensity in DMSO-treated siblings, *prpf4*^−/−^ embryos, and ATMi-treated *prpf4*^−/−^ embryos. Scale bar: 100 µm. **J** Quantitative analysis of o-dianisidine staining intensity. ATMi-treated *prpf4*^−/−^ embryos show significantly higher staining intensity compared to DMSO-treated *prpf4*^−/−^ embryos. **K**, **L** Effect of *p53* knockdown on erythrocyte hemoglobin levels in *prpf4*^−/−^ embryos at 48 hpf. **K** Knockdown of *p53* in *prpf4*^−/−^ embryos significantly increased hemoglobin levels, as indicated by stronger o-dianisidine staining. Scale bar: 200 µm. **L** Quantitative analysis of o-dianisidine staining intensity.

Splicing proteins prevent the formation of RNA:DNA hybrids (R-loops) by binding to nascent transcripts, thereby ensuring proper DNA replication. Depletion of splicing proteins may lead to R-loop accumulation and genome instability, subsequently activating the DNA damage response (DDR), which triggers cell cycle arrest and apoptosis [18, 19]. Given these findings, we hypothesized that *prpf4* deficiency might result in increased R-loop formation and DNA damage, thereby disrupting cell cycle progression and promoting apoptosis during erythropoiesis. To test this hypothesis, we assessed the protein levels of S9.6, a marker of R-loops [20], and γ-H2AX (phospho-Histone H2A.X), an early marker of DNA double-strand breaks (DSBs) [21]. The results show that the protein levels of both S9.6 and γ-H2AX were significantly elevated in *prpf4*^*−/−*^ embryos (Fig. 3A, B; Gel Supplementary). Immunofluorescence staining further confirmed a strong γ-H2AX signal in erythrocytes of *prpf4*^*−/−*^ embryos (Fig. 3C-E). These results demonstrate that *prpf4* deficiency induces DNA damage in erythrocytes.

P53 has been regarded as a key regulator of the DDR [22], orchestrating cell cycle arrest, DNA damage repair, and apoptosis by modulating downstream factors such as *p21*, *mdm2*, *cyclin G1*, *ccnh*, and *pik3r3a* [23]. Western blot analysis revealed that P53 protein was significantly elevated in *prpf4*^*−/−*^ embryos (Fig. 3F; Gel Supplementary). RT-qPCR analyses revealed that the mRNA levels of *p53* and its target genes *p21*, *cyclin G1*, *mdm2*, *ccnh*, and *pik3r3a* were significantly upregulated (Fig. S6D), indicating the enhanced activation of the p53 pathway.

During the DDR, ATM, and ATR regulate the p53 signaling pathway by promoting the phosphorylation of Chk2 and Chk1, respectively [24, 25], thereby coordinating cell cycle arrest, DNA repair, and apoptosis initiation [26]. To investigate the involvement of these two pathways in *prpf4*^*−/−*^ embryos, we evaluated the expression of phosphorylated Chk1 (p-Chk1) and Chk2 (p-Chk2), the downstream effectors of ATR and ATM, respectively. The results show that no significant change was observed in p-Chk1 protein levels in *prpf4*^*−/−*^ embryos (Fig. 3G; Gel Supplementary), whereas p-Chk2 protein levels were significantly elevated (Fig. 3H; Gel Supplementary), indicating that the ATM/Chk2 pathway was activated in *prpf4*^*−/−*^ embryos. These findings suggest that activation of the ATM/Chk2 pathway may contribute to upregulation of the p53 pathway and the resulting impairment of erythropoiesis. To validate this hypothesis, we treated *prpf4*^*−/−*^ embryos at 48 hpf with the ATM inhibitor KU60019 or the ATR inhibitor AZ20 [27] to suppress ATM and ATR pathway activity, respectively, and then analyzed erythropoiesis using o-dianisidine staining. The results show that the erythropoiesis defects were partially rescued in *prpf4*^*−/−*^ embryos treated with ATM inhibitor (Fig. 3I, J), whereas ATR inhibition had no observable effect (Fig. S7A, B). Additionally, blocking the p53 pathway by injecting *p53* MO also partially rescued the erythropoiesis defects in *prpf4*^*−/−*^ embryos (Fig. 3K, L). Collectively, these data suggest that *prpf4* deficiency induces DNA damage in erythrocytes, and the erythropoietic defects in *prpf4*^*−/−*^ embryos are at least partially mediated through activation of the ATM/Chk2–p53 signaling pathway, though additional p53-independent mechanisms may also contribute.

### DNA Damage-Induced ATM/Chk2-p53 Pathway Partially Mediates Erythrocyte Defects in *prpf4* Mutants

**Fig. 4:**
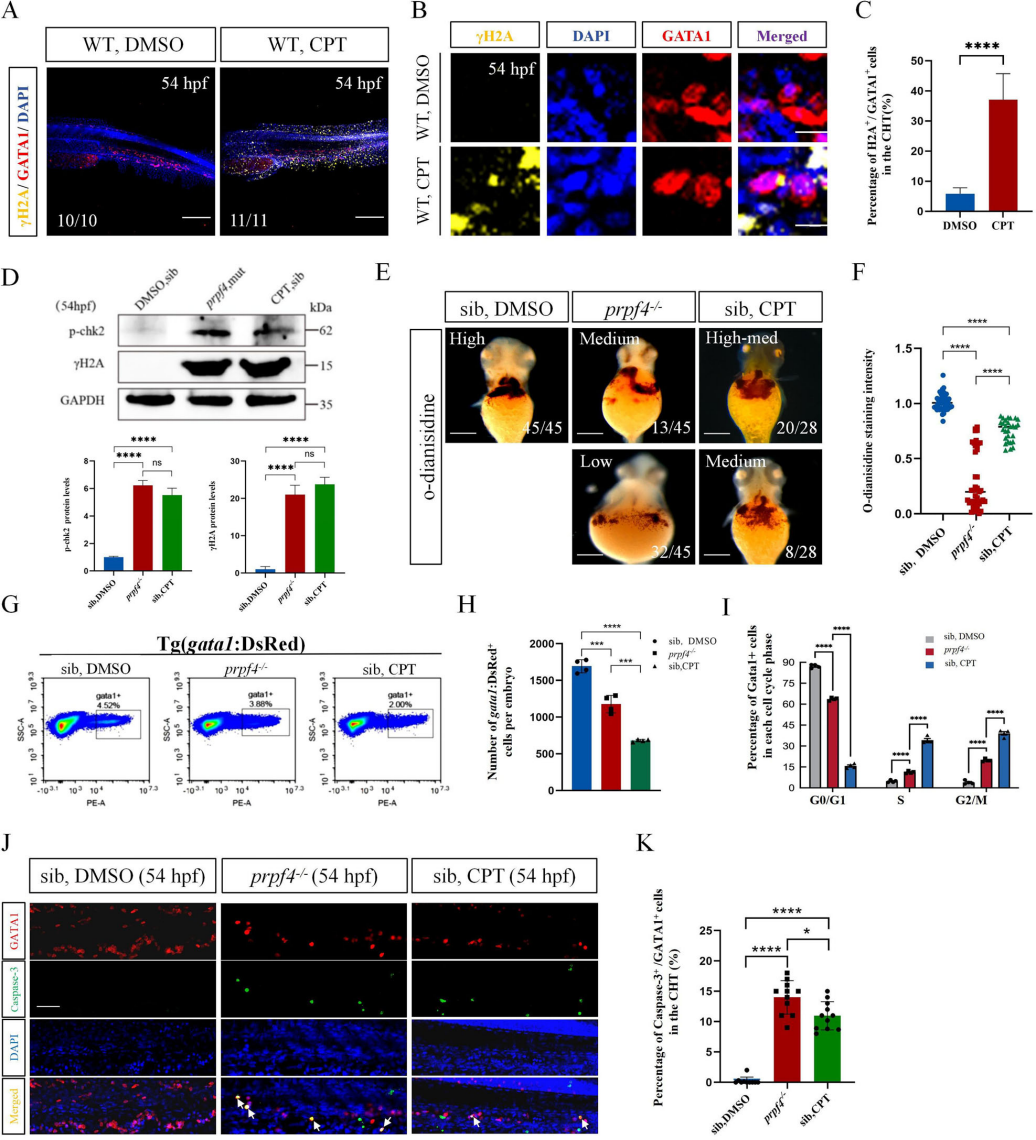
DNA damage-induced ATM/Chk2-p53 pathway partially mediates erythrocyte defects in *prpf4* mutants. **A-C** DNA damage examination in erythrocytes in the CHT region at 54 hpf. **A** Confocal images showing γH2AX (yellow) staining in WT (DMSO) and WT embryos treated with camptothecin. Scale bar: 200 µm. **B** High-magnification images of γH2AX and GATA1 colocalization in erythrocytes. Scale bar: 50 µm. **C** Quantitative analysis of γH2AX-positive cells among GATA1-positive cells in the CHT region. **D** Western blot analysis of p-Chk2 and γH2AX levels in 54 hpf embryos. Densitometric quantification of p-Chk2 and γH2AX protein levels is shown below. **E**, **F** Hemoglobin levels in 60 hpf embryos assessed by o-dianisidine staining. **E** Hemoglobin staining intensity in siblings treated with DMSO, *prpf4*^−/−^ embryos, and camptothecin-treated siblings. Staining intensity is categorized as high, medium, or low. Scale bar: 100 µm. **F** Quantitative analysis of o-dianisidine staining intensity. **G–I** FACS analysis of GATA1^+^ erythrocytes in Tg(*gata1*:DsRed) embryos at 54 hpf. **G** The percentage of GATA1^+^ cells in DMSO-treated siblings, *prpf4*^−/−^ embryos, and camptothecin-treated siblings (30 embryos per group). **H** Quantification of the total number of GATA1^+^ cells per embryo. **I** Cell cycle distribution of GATA1^+^ cells. *Prpf4*^−/−^ embryos and camptothecin-treated siblings exhibit cell cycle arrest. **J**, **K** Detection of apoptosis in GATA1^+^ erythrocytes in the CHT region of 54 hpf embryos. **J** Confocal microscopy images showing active Caspase-3 staining in DMSO-treated siblings, *prpf4*^−/−^ embryos, and camptothecin-treated siblings. Merged images highlight apoptotic signals (arrows) in GATA1^+^ cells. Scale bar: 50 µm. **K** Quantification of the percentage of active Caspase-3^+^ cells among GATA1^+^ cells in the CHT region. Both *prpf4*^−/−^ embryos and camptothecin-treated siblings show a significant increase in apoptosis compared with DMSO-treated siblings.

Our data have shown that *prpf4* mutant leads to DNA damage and upregulation of the ATM/Chk2-p53 cascade, and that this upregulation partially contributes to the erythropoietic defects in *prpf4*^*−/−*^ embryos. However, we could not directly conclude that *prpf4* mutation results in DNA damage, which sequentially upregulates the ATM/Chk2-p53 cascade and disturbs erythropoiesis. Camptothecin (CPT), a topoisomerase I inhibitor, induces the DDR by activating ATM kinase through the induction of DNA double-strand breaks (DSBs) [28]. To further investigate the role of DNA damage in erythrocyte cell cycle arrest and apoptosis during erythropoiesis, we treated embryos with CPT to mimic the DNA damage observed in the erythroid progenitors of *prpf4*^*−/−*^ embryos and analyzed erythropoiesis. Immunofluorescence analysis revealed that γH2AX levels were significantly elevated in CPT-treated embryos compared to controls (Fig. 4A), and GATA1^+^ cells in CPT-treated embryos exhibited markedly increased γH2AX staining (Fig. 4B, C). Western blot analysis further confirmed that CPT treatment significantly increased the protein levels of γH2AX and p-Chk2, which were comparable to those observed in *prpf4*^*−/−*^ embryos (Fig. 4D; Gel Supplementary). In addition, o-dianisidine staining showed that hemoglobin levels were significantly reduced in CPT-treated embryos relative to untreated siblings, but remained higher than those in *prpf4*^*−/−*^ embryos (Fig. 4E, F). To gain further insights into the effects of DDR on erythrocyte growth and survival, we analyzed the number of erythrocytes in controls, CPT-treated, and *prpf4*^*−/−*^ embryos at 54 hpf using flow cytometry. The results show that the number of GATA1^*+*^ cells was significantly reduced in CPT-treated embryos compared to controls (Fig. 4G, H), accompanied by cell cycle arrest at the S and G2/M phases (Fig. 4I). Furthermore, similar to *prpf4*^*−/−*^ embryos, the number of caspase-3-positive cells in the CHT region was significantly increased in CPT-treated embryos compared to controls (Fig. 4J, K). These findings further support the notion that *prpf4* deficiency induces DDR, which contributes to defective erythropoiesis.

Notably, compared to *prpf4*^*−/−*^ embryos, CPT-treated embryos exhibited a more pronounced reduction in GATA1^+^ cells (Fig. 4G, H) and more severe cell cycle arrest at the S and G2/M phases (Fig. 4I). However, the reduction in o-dianisidine staining intensity was less severe in CPT-treated embryos than in *prpf4*^*−/−*^ embryos (Fig. 4E, F). These results suggest that although *prpf4*^*−/−*^ embryos display milder cell cycle arrest in erythrocytes and retain more erythrocytes compared to CPT-treated embryos, they exhibit a more pronounced deficiency in o-dianisidine staining, implying that *prpf4* deficiency may impair the process of erythrocyte maturation in an alternative way.

### The Maturation of Erythrocytes Is blocked in *prpf4* Mutants

**Fig. 5:**
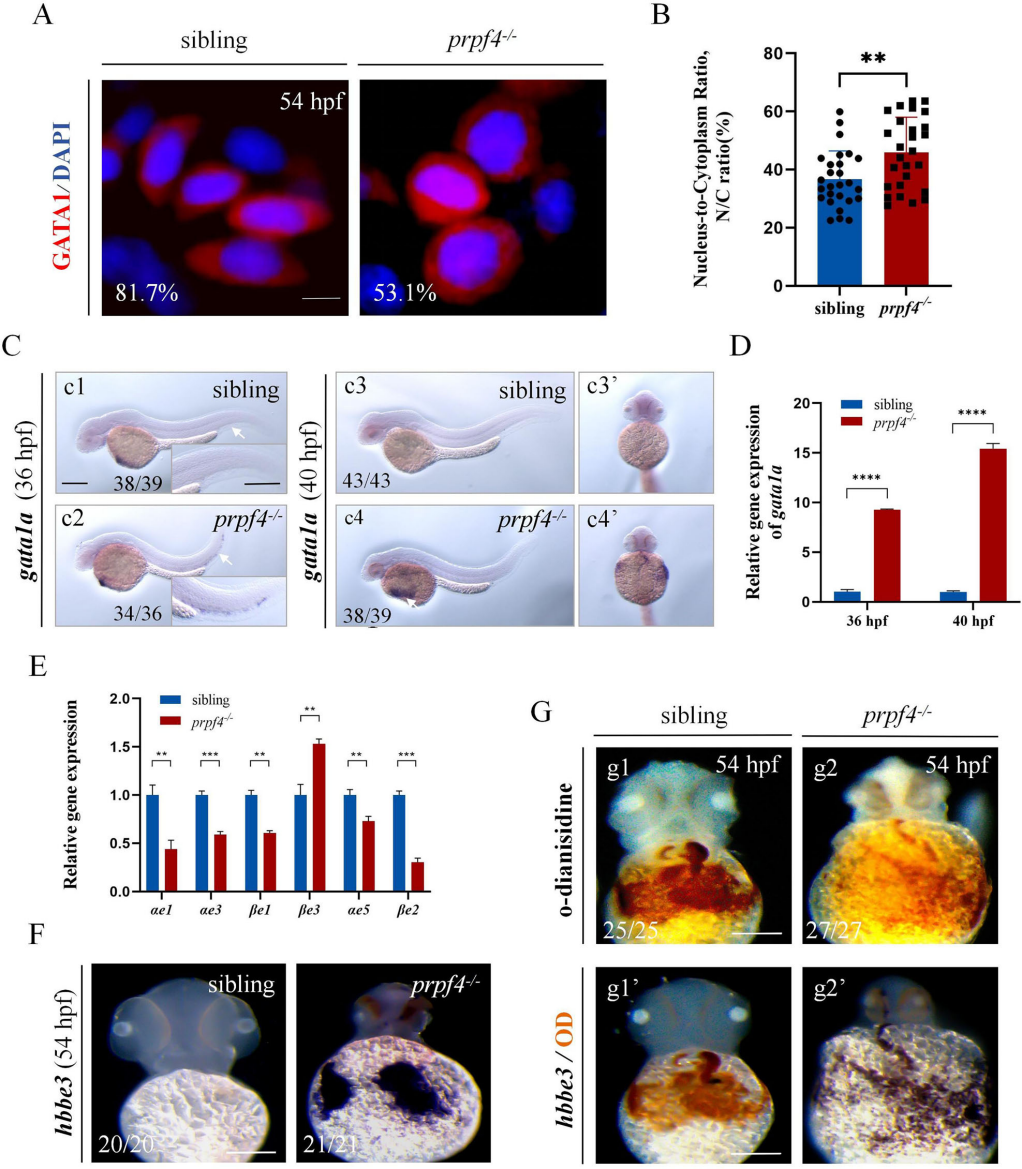
The maturation of erythrocytes is disturbed in *prpf4* mutants. **A**, **B** Morphological analysis of erythrocytes in siblings and *prpf4*^−/−^ embryos at 54 hpf. **A** Representative confocal images showing GATA1^+^ cells stained with DAPI in siblings and *prpf4*^−/−^ embryos. In sibling embryos, 81.7% of erythrocytes exhibit an oval morphology, while in *prpf4*^−/−^ embryos, 53.1% of erythrocytes display a round morphology. The presented images represent typical erythrocyte morphologies observed in each group. Scale bar: 20 µm. **B** Quantification of the nuclear-to-cytoplasmic (N/C) ratio of individual GATA1^+^ cells (30 embryos per group). **C** WISH analysis of *gata1a* expression in siblings and *prpf4*^−/−^ embryos at 36 hpf and 40 hpf. Lateral views (**c3, c4**) and ventral views (**c3’, c4’**). Scale bar: 200 µm. **D** Relative expression of *gata1a* in *prpf4*^−/−^ mutants and siblings at 36 hpf and 40 hpf, as determined by RT-qPCR. **E** RT-qPCR analysis of erythroid-related gene expression in siblings and *prpf4*^−/−^ embryos. **F** WISH analysis of *hbbe3* expression in siblings and *prpf4*^−/−^ embryos at 54 hpf. G Combined analysis of *hbbe3* expression and hemoglobin levels in embryos. **g**1**, g**2 O-dianisidine staining reveals reduced hemoglobin levels in *prpf4*^−/−^ embryos compared to siblings. **g**1′, **g**2′ Whole-mount in situ hybridization for *hbbe3* performed on the same embryos as in (**g**1) and (**g**2). Scale bars: 100 µm (**F,** **G**).

During erythrocyte maturation, cell volume initially increases and then decreases, accompanied by progressive nuclear condensation [9]. Therefore, the nuclear-to-cytoplasmic (N/C) ratio could be used as an indicator for erythrocyte maturation. To assess erythrocyte maturation, we analyzed the morphology of erythrocytes at 54 hpf using confocal microscopy. Compared to siblings, erythrocytes in *prpf4*^*−/−*^ embryos appeared larger and more rounded (Fig. 5A; Fig. S7C, D), with a significantly increased N/C ratio (Fig. 5B). Additionally, we observed increased *gata1a* expression in the CHT region of *prpf4*^*−/−*^ embryos at 36 hpf compared to siblings (Fig. 5C, c1-c2, arrows). At 40 hpf, *gata1a* expression was no longer detected in circulating erythrocytes of control embryos, while it remained highly expressed in *prpf4*^*−/−*^ embryos (Fig. 5C, c3-c4’, arrow). Consistently, RT-qPCR analysis also showes a significant upregulation of *gata1a* mRNA at both 36 and 40 hpf (Fig. 5D). These findings suggest that erythrocyte maturation is impaired in *prpf4*^*−/−*^ embryos.

During erythropoiesis, the expression of embryonic globin *αe1*, *αe3*, *βe1*, and *βe3* begins at the somitogenesis stage, while the expression of *αe5* becomes significantly upregulated only at 14 dpf [29]. Among these, *βe3* levels decrease between 24-48 hpf and are nearly absent from circulating erythrocytes after 48 hpf [30]. RT-qPCR analysis revealed that, compared to siblings, the expression of *αe1*, *αe3*, *αe5*, *βe1*, and *βe2* were significantly reduced in *prpf4*^*−/−*^ embryos, while *βe3* expression remained abnormally high (Fig. 5E). Consistently, in situ hybridization at 54 hpf showed that *βe3* expression was undetectable in erythrocytes within the ventral yolk sac region of siblings, but remained strongly expressed in the same region in *prpf4*^*−/−*^ embryos (Fig. 5F). These findings further indicate that erythrocyte maturation is blocked in *prpf4*^*−/−*^ embryos. To directly examine the relationship between *hbbe3* expression and erythrocyte maturation within individual embryos, we sequentially performed the o-dianisidine staining and in situ hybridization for *hbbe3* in the same embryos [9]. The results show that in *prpf4*^*−/−*^ embryos, *hbbe3* was expressed in regions exhibiting weak o-dianisidine staining in the ventral yolk sac region, whereas in siblings, *hbbe3* expression was absent from areas showing strong o-dianisidine signals(Fig. 5G). These findings further demonstrate that a subset of erythrocytes in *prpf4*^*−/−*^ embryos fails to complete normal maturation.

### *prpf4* Deficiency Causes Aberrant Splicing of Genes Related to Erythropoiesis

**Fig. 6:**
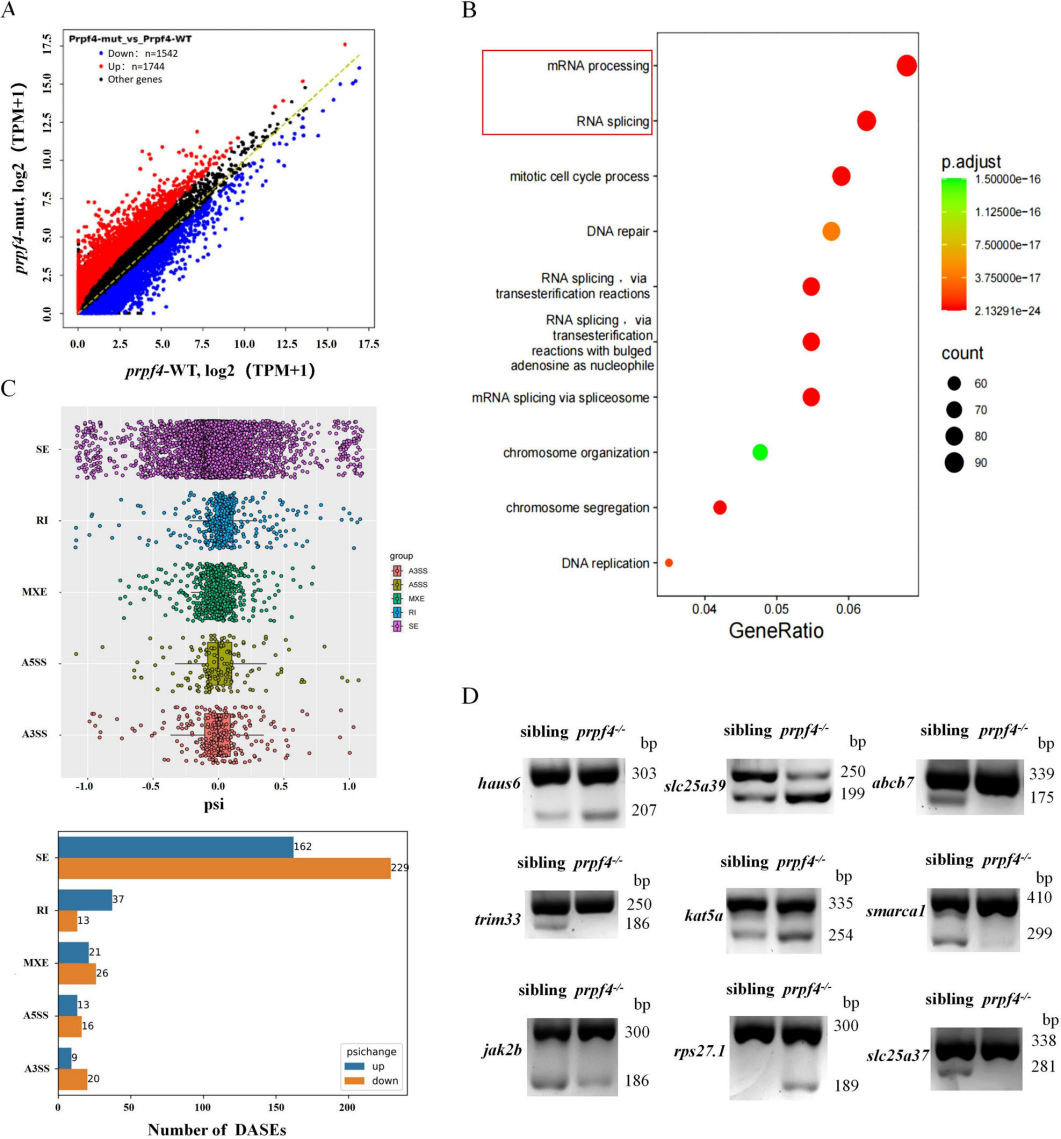
*prpf4* deficiency leads to aberrant splicing of genes related to erythrocyte differentiation and maturation. **A** RNA-seq analysis reveals significant differential gene expression in *prpf4*^−/−^ mutants compared to siblings. Differentially expressed genes were identified using a fold-change threshold and adjusted *p*-value. **B** Gene Ontology (GO) enrichment analysis of upregulated genes in *prpf4*^−/−^ mutants. **C** Differential alternative splicing events (DASEs) in *prpf4*^−/−^ mutants. PSI (Percent Spliced-In) indicates the proportion of transcripts including a given exon; changes in PSI reflect shifts in splicing patterns. Splicing events with FDR < 0.05 and |ΔPSI | ≥ 0.1 were considered significant. Top: Scatter plot of PSI (Percent Spliced-In) changes for different types of splicing events, including skipped exon (SE), retained intron (RI), mutually exclusive exon (MXE), alternative 5’ splice site (A5SS), and alternative 3’ splice site (A3SS). SE events are the most affected, with a predominance of negative PSI changes. Bottom: Bar plot showing the number of upregulated and downregulated DASEs for each splicing event type. The majority of DASEs are SE events, with 229 downregulated and 162 upregulated cases. **D** Validation of aberrant splicing events in erythropoiesis-related genes by SqRT-PCR at 48 hpf. Differential splicing patterns were confirmed in *prpf4*^−/−^ embryos for representative genes including *haus6*, *slc25a39*, *abcb7*, *trim33*, *kat5a, smarca1, jak2b*, *rps27.1*, and *slc25a37*.

*PRPF4* is an essential component of the U4/U6-U5 tri-snRNP complex, which plays a critical role in pre-mRNA splicing [31]. We hypothesized that defective alternative splicing caused by *prpf4* mutation might contribute to defects in erythropoiesis. To test this hypothesis, we performed RNA-seq analysis on sorted erythrocytes from *prpf4*^*−/−*^ embryos and siblings. The results revealed that, compared to siblings, 1744 genes were upregulated and 1542 genes were downregulated in *prpf4*^*−/−*^ embryos (Fig. 6A and Table S2). The upregulated genes were significantly associated with mRNA processing, pre-mRNA splicing, cell cycle, and DNA damage repair (Fig. 6B). Given that mRNA processing and pre-mRNA splicing factors exhibited the most significant changes (as indicated by the red box), we further conducted differential alternative splicing analysis. Among the 7135 alternative splicing events (Table S3), which corresponded to 3450 unique genes, 546 events (involving 435 unique genes) showed significant differences (Fig. 6C; Table S4), accounting for 7.65%. Most of these events (71.61%) were of the skipped exon (SE) type, with approximately 58.57% exhibiting negative percent spliced-in (PSI) values and 41.43% showing positive PSI values (Fig. 6C). These findings indicate that *prpf4* deficiency primarily affects selective alternative splicing events (exon skipping (SE), rather than causing global splicing defects (only a smaller number of RI, A5SS, A3SS, and MXE events were affected). Through GO functional annotation of these differentially spliced genes, we identified three categories directly associated with erythrocyte development: heme biosynthetic process (GO:0006783; including *slc25a39*, *abcb7*, *uros, abcb10*, and *hmbsa*), erythrocyte differentiation (GO:0030218; including *trim33*, *jak2b*, *rps27.1*, and *slc25a37*), and primitive erythrocyte differentiation (GO:0060319; including *smarca1*, *ash2l*, and *kat5a*). To further validate that *prpf4* mutation disrupts alternative splicing of these erythropoiesis-related regulatory genes, we performed SqRT-PCR for the differential splicing events in erythrocytes sorted from siblings and *prpf4*^*−/−*^ embryos at 48 hpf. The results confirm that the erythropoiesis-related genes exhibited aberrant alternative splicing patterns in *prpf4*^*−/−*^ embryos (Fig. 6D). These findings demonstrate that *prpf4* mutation leads to aberrant splicing of certain genes involved in erythropoiesis.

### *slc25a39* Downregulation in *prpf4* Mutants Blocks Erythrocyte Maturation

**Fig. 7:**
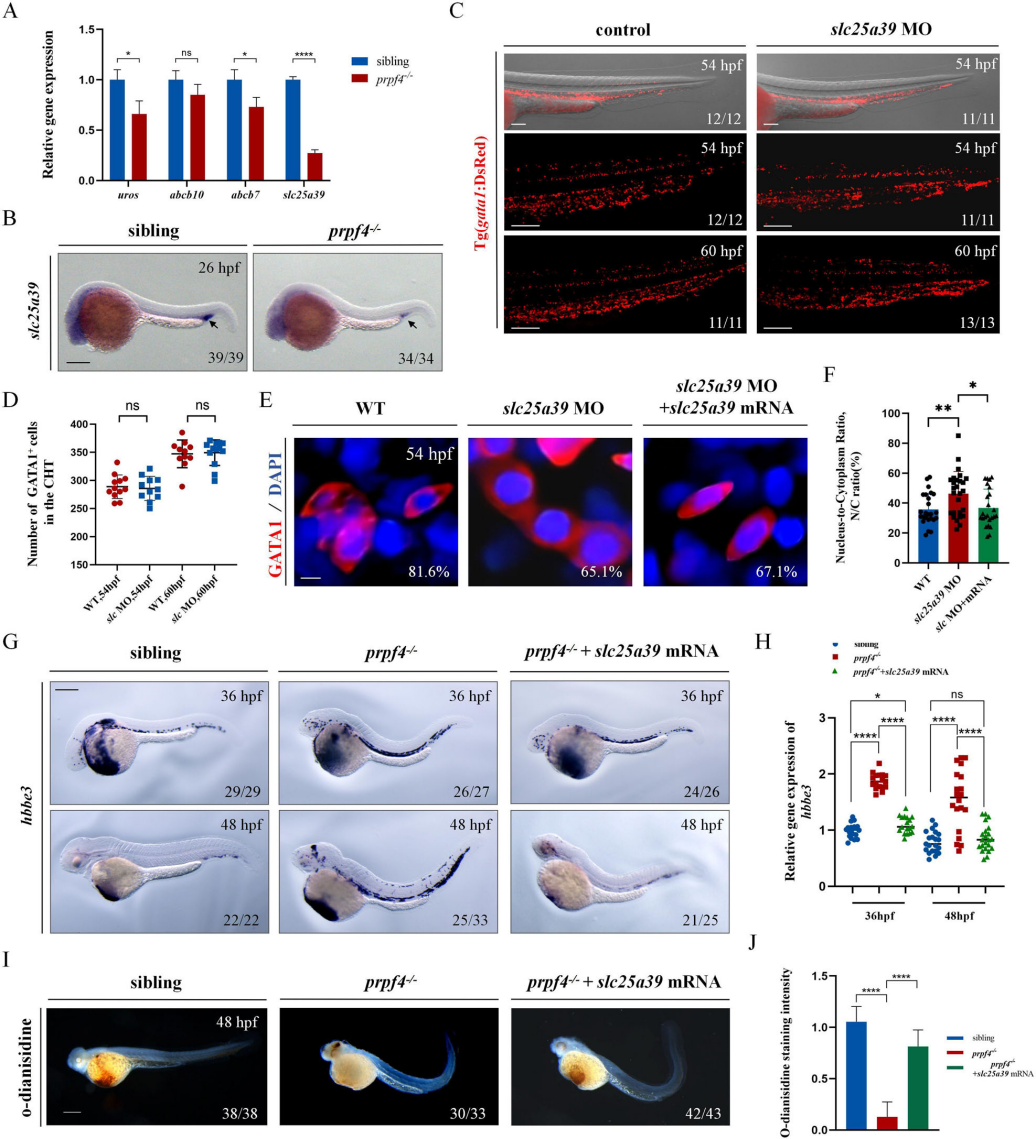
*slc25a39* downregulation in *prpf4* mutants blocks erythrocyte maturation. **A** RT-qPCR analysis of gene expression in GATA1^+^ erythrocytes at 48 hpf. **B** Expression of *slc25a39* in siblings and *prpf4*^−/−^ embryos at 26 hpf, detected by in situ hybridization. Scale bar: 200 µm. **C**, **D** Effect of *slc25a39* knockdown on GATA1^+^ erythrocytes in Tg(*gata1*:DsRed) embryos. **C** Confocal images showing GATA1^+^ cells in the CHT region of control and *slc25a39* MO-injected embryos at 54 hpf and 60 hpf. Scale bar: 100 µm. **D** Quantification of the number of GATA1^+^ cells in the CHT region. **E**, **F** Effect of *slc25a39* knockdown on erythrocytes morphology in Tg(*gata1*:DsRed) embryos at 54 hpf. **E** Confocal images showing the morphology of GATA1^+^ cells in control, *slc25a39* MO-injected, and slc25a39 MO + mRNA co-injected embryos. Scale bar: 20 µm. **F** Quantitative analysis of the nuclear-to-cytoplasmic (N/C) ratio of individual cells measured from (**E**). WT (*n* = 25), *slc25a39* MO (*n* = 27), *slc25a39* MO + mRNA (*n* = 24). **G** WISH analysis of *hbbe3* at 36 hpf shows the staining patterns in sibling, *prpf4*^−/−^, and *prpf4*^−/−^ embryos co-injected with *slc25a39* mRNA. Scale bar: 200 µm. **H** Quantification of *hbbe3* staining intensity in the CHT region at 36 hpf reveals significantly higher staining intensity in *prpf4*^−/−^ embryos compared with sibling embryos. Co-injection of *slc25a39* mRNA significantly reduces the staining intensity in *prpf4*^−/−^ embryos. **I**, **J** Effect of *slc25a39* mRNA injection on hemoglobin levels in *prpf4*^−/−^ embryos at 48 hpf. **I** Images of o-dianisidine staining in WT, *prpf4*^−/−^, and *prpf4*^−/−^ embryos injected with *slc25a39* mRNA. Scale bar: 200 µm. **J** Quantification of o-dianisidine staining intensity. Hemoglobin levels are significantly reduced in *prpf4*^−/−^ embryos compared with WT, while *slc25a39* mRNA injection partially restores hemoglobin levels in *prpf4*^−/−^ embryos. Scale bar: 100 µm.

Mutations or functional knockdown of certain heme synthesis-related genes could impair erythrocyte maturation and result in the loss of o-dianisidine staining during erythrocyte maturation [32]. Since our data have demonstrated that *prpf4* mutations lead to impaired erythrocyte maturation (Fig. 5), we performed RT-qPCR to further analyze the expression of heme synthesis-related genes with RNA splicing defects in *prpf4*^*−/−*^ embryos. The results revealed decreased expression of *uros*, *abcb10*, *abcb7*, and *slc25a39*, with *slc25a39* showing the most significant reduction (Fig. 7A). Consistently, in situ hybridization demonstrated that *slc25a39* expression was markedly reduced in *prpf4* mutant embryos at 26 hpf (Fig. 7B). Previous studies have reported that loss of function of *slc25a39* impairs erythropoiesis and eliminates o-dianisidine staining in zebrafish embryos [32]. Our data similarly show that *slc25a39* knockdown impaired erythrocyte maturation but did not affect erythrocyte survival. Although no obvious morphological abnormalities were observed in *slc25a39* morphants, the circulating blood in the heart region appeared paler than in controls (Fig. S8A). The expression of *scl*, *c-myb, gata1a, pu.1*, and *lyz* was intact (Fig. S8B, C); however, o-dianisidine staining was greatly diminished (Fig. S8D). Additionally, at later stages, the number of erythrocytes in the CHT remained unchanged in *slc25a39* morphants (Fig. 7C, D), but the erythrocytes appeared more rounded and displayed increased nuclear-to-cytoplasmic (N/C) ratios (Fig. 7E, F; Fig. S9A, B). The expression of *hbbe3* was also elevated at 48 hpf and 60 hpf (Fig. S9C, D). Given these findings, we hypothesized that reduced *slc25a39* expression contributes to erythrocyte maturation defects in *prpf4*^*−/−*^ embryos. Indeed, injection of *slc25a39* mRNA into *prpf4*^*−/−*^ embryos partially rescued *hbbe3* expression (Fig. 7G, H) and o-dianisidine staining (Fig. 7I, J), although it did not restore the external phenotypes such as microcephaly and microphthalmia (Fig. 7G-J). These results suggest that *slc25a39* splicing defects, leading to reduced *slc25a39* mRNA levels, contribute to the erythrocyte maturation defects observed in *prpf4*^*−/−*^ embryos.

## Discussion

Dysfunction of *Prpf4* has been reported to play critical roles in both development and disease. During embryonic development, *Prpf4* knockdown impairs the pluripotency, proliferation, and differentiation of mESCs [15], and *prpf4* mutations disrupt the migration of pLLP cells in zebrafish [16]. In breast, colon, and lung cancers, overexpression of *PRPF4* significantly promotes cell proliferation, invasion, and migration [33, 34]. Additionally, *PRPF4* contributes to the reversal of cancer cell death induced by anti-cancer drugs by regulating actin cytoskeleton remodeling and epithelial-mesenchymal transition (EMT), highlighting its potential as a novel therapeutic target for cancer [35]. GEPIA further shows that *PRPF4* is highly expressed in DLBC (Fig. S1A), and patients with high *PRPF4* expression had significantly lower survival rates compared to those with low expression (Fig. S1B), suggesting a potential role of *PRPF4* in hematopoiesis. More importantly, data from the BloodSpot database show that mouse *Prpf4* expression is most enriched in the CFU-E cells (Fig. S1C), further implying the critical role of *Prpf4* in erythropoiesis. Here, using zebrafish embryos, we identified that *prpf4* plays crucial roles during erythropoiesis.

During late stages of erythropoiesis, thousands of pre-mRNAs must be precisely and efficiently spliced to ensure proper erythrocyte maturation. Cell and animal models harboring splicing factor mutations have demonstrated the essential roles of splicing factors in hematopoietic cell fate determination and differentiation. Different spliceosomal components play distinct roles in hematopoiesis, with varying lineage sensitivities. For example, *SF3B1* mutations in myelodysplastic syndromes specifically impair the erythroid lineage by inducing mis-splicing of *MAP3K7*, which leads to p38 MAPK inactivation and premature downregulation of *GATA1*, thereby blocking terminal erythroid differentiation [7]. In zebrafish, *Sf3b1* regulates erythroid maturation and proliferation through TGFβ signaling [9]. *U2af1* is essential for the survival and function of HSPCs [10]. *Sart3* deficiency disrupts HSPC development via the p53 pathway in zebrafish [36]. In mice, physiological expression of the *Srsf2* P95H mutation causes HSPC dysfunction and aberrant RNA splicing [37]. Recently, forward genetic screens have identified dozens of zebrafish mutant lines carrying mutations in splicing factor genes, highlighting both conserved and unique roles of different splicing factors in hematopoiesis [9, 24, 36, 38–42]. In this study, we demonstrate that *prpf4* mutations primarily affect erythrocyte proliferation and maturation during definitive hematopoiesis. Similar to other splicing factors [38], *prpf4* mutations result in erythrocyte cell cycle arrest and apoptosis. In addition, *prpf4* deficiency disrupts the splicing of *slc25a39* pre-mRNA, leading to a marked reduction in *slc25a39* mRNA levels and impaired erythrocyte maturation. This dual role of *prpf4* in regulating both erythrocyte proliferation and maturation has not been previously reported and provides new insights into the complex mechanisms by which RNA splicing factors control erythropoiesis. Here we also found that *abcb7* and *abcb10* were slightly downregulated in *prpf4* mutants (Fig. 7A). Given that ABCB7 dysfunction has been linked to hematologic disorders, in which aberrant splicing of ABCB7 disrupts mitochondrial iron homeostasis [43]. In addition, ABCB10 also forms a complex with SLC25A37 and ferrochelatase to promote mitochondrial iron utilization for heme biosynthesis during erythropoiesis [44]. Although our data show that downregulation of *slc25a39* greatly contributes to erythrocyte maturation defects observed in *prpf4* mutants, we cannot exclude the potential involvement of other heme biosynthesis-related genes in this process.

Currently, two non-mutually exclusive hypotheses have been proposed to explain the different roles of RNA splicing factors in hematopoiesis [45]: (1) Different spliceosomal components may regulate distinct downstream targets due to variations in splice site recognition and splicing patterns. (2) Some phenotypic differences may arise from the non-spliceosomal functions of individual splicing factor. Besides canonical splicing, several splicing factors have been shown to exhibit non-spliceosomal functions in diverse cellular processes. The splicing factor SRSF1 interacts with the RPL5–MDM2 complex to prevent p53 degradation and induce senescence [46], while SF3A2 and Prp31 regulate spindle–centromere–Ndc80 interactions to ensure proper chromosome segregation during mitosis [47]. Moreover, accumulating evidence suggests that splicing factor mutations may induce genomic instability through R-loop accumulation. For instance, mutations in SRSF2 or U2AF1 lead to R-loop–associated activation of PARP1 and sensitize cells to PARP inhibitors [48]. Similarly, SRSF2 and SF3B1 mutations cause R-loop–induced replication stress and ATR pathway activation, ultimately resulting in DNA damage and apoptosis [49, 50]. Here, our study reveals that *prpf4* deficiency leads to excessive R-loop formation and DNA damage in erythrocytes, which partially results in cell cycle arrest and apoptosis. To further validate this mechanism, we treated embryos with CPT and found that CPT treatment induced DNA damage-ATM/Chk2-p53 signaling pathway, blocked cell cycle progression, and increased apoptosis in erythrocytes (Fig. 4). These findings confirm that DNA damage induced by *prpf4* deficiency contributes to erythropoietic defects, and further imply that disruption of other splicing factors may also impair erythropoiesis through similar mechanisms. Notably, pre-mRNA splicing is crucial for overall system development. We also observed the overall developmental defects, such as a smaller head and a curved tail at the later stage in *prpf4* mutants (Fig. 1B, b1). Therefore, we cannot exclude the possibility that other developmental processes, such as hematopoietic niches, also influence hematopoiesis during definitive hematopoiesis.

In previous study, it was reported that *prpf31* deficiency extensively perturbs the alternative splicing of mitosis-related genes, resulting in aberrant mitosis and M phase arrest during HSPC expansion [51]. In our RNA-seq data, we also observed altered expression of several mitosis-related genes (Fig. 6B), suggesting that *prpf4* may regulate mitotic gene splicing directly to ensure proper erythrocyte cell cycle progression. Therefore, the DNA damage is more likely to arise from R-loop accumulation and specific signaling defects.

Notably, at 26 hpf, the expression levels of *gata1a* and *hbbe3* were slightly elevated in *prpf4*^*−/−*^ embryos compared to controls (Fig. S4B, C). This transient upregulation may reflect an early compensatory response within the erythroid lineage prior to the onset of terminal differentiation defects. Alternatively, it may result from the accumulation of immature erythroid precursors due to impaired progression toward maturation. At 48 hpf, the expression of the immature erythrocyte marker *hbbe3* was elevated in the ventral yolk sac region of *prpf4*^*−/−*^ embryos, while it was partially reduced in the CHT region (Fig. 1R). These data suggest that the maturation of most erythrocytes in the ventral yolk sac region is inhibited in *prpf4* mutants (Fig. 5E-G), whereas a significant portion of erythrocytes in the CHT region undergo cell cycle arrest and apoptosis (Fig. 2G, H; Fig. S6B, C). In addition, erythrocyte maturation is also impaired in the CHT region, as evidenced by altered morphology and increased N/C ratios (Fig. 5A, B). Although *prpf4* primarily regulates erythropoiesis during hematopoiesis, our findings also indicate that *prpf4* deficiency results in a mild defect in HSPCs development at later definitive hematopoiesis, as evidenced by a slight reduction in C-myb^+^ cells in the CHT region at 48 hpf in *prpf4*^*−/−*^ embryos (Fig. S5D, E). This mild phenotype is in contrast to that observed in *sf3b1* and *prpf31* zebrafish mutants [9, 51], in which the number of HSPCs was greatly decreased. Therefore, it is plausible that the modest decrease in HSCs in *prpf4* mutants may partially contribute to the reduced erythrocyte output observed at later stages.

Although the roles of RNA splicing factors, including *Prpf4*, are complicated and varied in hematopoiesis, our study does not fully elucidate the functions of *prpf4* in this process. Nevertheless, our findings reveal two critical roles of *prpf4* during erythropoiesis: regulating early erythrocyte proliferation and ensuring late-stage erythrocyte maturation. On one hand, *prpf4* mutations lead to the accumulation of R-loops and DNA damage, thereby activating the ATM/Chk2-p53 signaling pathway, which induces cell cycle arrest and apoptosis in early erythrocytes. On the other hand, *prpf4* mutations disrupt pre-mRNA splicing during erythrocyte maturation, resulting in a significant reduction in mature *slc25a39* mRNA, impaired hemoglobin synthesis, and defective erythrocyte maturation. Although our study demonstrates this dual function of *prpf4* in erythropoiesis, it does not address its potential role in HSPCs development. Further investigation is required to clarify the detailed roles of *prpf4* in hematopoiesis. Overall, our current data suggest that, during erythropoiesis, the normal function of *prpf4* is essential for maintaining the genomic stability of erythrocytes to ensure normal cell cycle progression, and is also crucial for erythrocyte maturation by regulating alternative splicing of *slc25a39* pre-mRNA.

## Methods and materials

### Ethics statement

All experimental protocols were approved by Chengdu Medical College (Sichuan, China). Zebrafish were maintained in accordance with the Guidelines of the Animal Care Committee of Chengdu Medical College.

### Fish maintenance

Wild type (AB), transgenic line Tg(*gata1*:DsRed) [52], Tg(*lyz*:DsRed) [53], *prpf4*^*t243*^ [16] fishes were maintained under standard conditions at about 28.5 °C. The developmental stages were characterized as previously described [54].

### Chemical treatment

As previously reported, the zebrafish embryos were treated with Camptothecin (50 nM, Beyotime, SC0141) [55], ATMi KU60019 (30 nM, Selleck, S1570) [38, 56] and ATRi AZ20 (30 nM, Sellleck, S7050) [38, 57]. In brief, the chemicals were diluted with egg water as the concentrations described above. The embryos were incubated with the chemicals from 24 hpf to the required stage.

### Plasmid construction

The total RNA was extracted following the manufacturer’s instruction (TRIzol, Ambion,15596-026). cDNA was prepared using Revert Aid First Strand cDNA Synthesis Kit (Fermentas, K1622) according to the manufacturer’s instructions. The full-length coding sequences (CDSs) of *prpf4* and *slc25a39* were amplified by PCR (PrimeSTAR Max Premix Takara, R045A) from zebrafish cDNA using gene-specific primers. Each primer pair was designed to include a 15 bp 5′ extension homologous to the ends of the linearized PCS^2+^ vector to enable seamless recombination and the PCS^2+^ vector was linearized by PCR using primers flanking the multiple cloning site. Cloning was performed using the In-Fusion HD Cloning Kit (Takara, 638909) following the manufacturer’s instructions. Briefly, 50–100 ng of insert and 100 ng of linearized vector were incubated with 2 μL 5× In-Fusion enzyme mix in a 10 μL total reaction volume at 50 °C for 15 min. The recombination products were transformed into Trelief 5α Chemically Competent Cells (Tsingke, TSC-C01), plated on LB/ampicillin plates, and positive clones were screened by colony PCR and confirmed by Sanger sequencing. The primers for cloning were listed in Table S1.

### MO and mRNA injection

Morpholino oligos (MO) for *slc25a39* (ATG MO, 5′-CTGCTTGTGATCTGTACCTTGAAAG-3′) [32], *p53* MO (ATG MO, 5′-GCGCCATTGCTTTGCAAGAATTG-3′) [58] and control MO (5′-CCTCTTACCTCAGTTACAATTTATA-3′) were obtained from Gene Tools. *Prpf4* mRNA and *slc25a39* mRNA were synthesized in vitro using mMESSAGE mMACHINE Kit (Ambion, AM1340). The concentration of MO was as follows: *slc25a39* MO, 300 μM; *p53* MO, 200 μM; control MO, 100 μM. The concentration for mRNA injection was as follows: *prpf4* mRNA, 30 ng/μl; *slc25a39* mRNA, 30 ng/μl. All the MOs and mRNAs were injected at the 1-4 cell stage.

### Cell cycle analysis

At 48 hpf, embryos (30 per tube) were rinsed 1–2 times with PBS, followed by the addition of 200 μL PBS containing 0.25% trypsin to each tube. Tissues were mechanically dissociated by pipetting for 5 min to obtain a homogeneous single-cell suspension. The samples were centrifuged at 500 × *g* for 5 min at 4 °C. The cell pellet was washed twice with PBS supplemented with 2% FBS. The digestion was then stopped by adding 500 μL PBS with 2% FBS and gentle pipetting for 5 min. The suspension was filtered through a cell strainer, centrifuged, and resuspended in 250 μL ice-cold PBS. To fix cells, 600 μL ice-cold absolute ethanol (final concentration: 70–75% ethanol) was added, mixed by inversion to prevent aggregation, and incubated at room temperature for 1 h. Fixed cells were collected by centrifugation at 300 × *g* for 5 min, rehydrated with 500 μL ice-cold PBS for 15 min, and stained with 500 μL DAPI working solution in the dark at room temperature for 15 min. Finally, cell cycle analysis was performed using a flow cytometer with appropriate fluorescence gating. DAPI excitation was achieved using a 355 nm ultraviolet laser, and emission was detected at a maximum wavelength of 500 nm.

### Erythrocytes sorting and RNA sequencing

To compare the transcriptomes of erythrocytes at 48 hpf, DsRed-labeled erythrocytes from siblings and *prpf4* mutants (on the Tg(*gata1*:DsRed) transgenic background) were isolated by flow cytometry (MoFlo XDP, Beckman). More than 10000 erythrocytes were sorted into 0.5% BSA-DPBS solution for each group. Double-stranded DNA (dsDNA) libraries were generated from the sorted cells according to a previously described protocol [59]. cDNA libraries were prepared using the TruePrep DNA Library Prep Kit V2 for Illumina (Vazyme, TD501). RNA sequencing was performed using the NovaSeq X Plus system by HaploX company. Cleaned FASTQ data were aligned to the zebrafish reference genome danRer11 using HISAT2. Raw counts of all RNA-seq samples were calculated by featureCounts from the SAM files generated by HISAT2. Normalized counts were generated by R packages of DESeq2 from raw counts files, and gplots and clusterProfiler R packages were used to analyze differentially expressed genes, and gene ontology (GO) enrichment as described previously [60]. Differentially expressed genes (DEGs) were defined as those with an absolute log2 fold change ≥1 and a false discovery rate (FDR) adjusted *p*-value < 0.05, calculated using the DESeq2 package.

### Semi-quantitative RT-PCR (sqRT-PCR) and quantitative RT-PCR (RT-qPCR)

Total RNA was extracted using TRIzol reagent (Invitrogen), and cDNA synthesis was performed using RevertAid First Strand cDNA Synthesis Kit (Thermo Fisher Scientific) according to the manufacturer’s instructions. For sqRT-PCR, PCR amplification was performed on the cDNA using specific primers. The PCR products were separated on a 2% agarose gel containing ethidium bromide and imaged using a Bio-Rad chemiluminescence imaging system. RT-qPCR was performed using the ChamQ Universal SYBR qPCR Master Mix (Vazyme, #Q711) and the CFX96 Real-Time System (BIO-RAD) according to manufacture’s instructions. Relative mRNA expression levels were calculated using the 2^−ΔΔCt^ method and normalized to *β-actin*. Primers are listed in Table S1. All experiments were performed in at least three independent biological replicates.

### Whole-mount in situ hybridization

In situ hybridization was performed as described in previous study [61]. The previous probes *scl, lyz, hbbe3, gata1, fli1a, pu.1, c-myb* were used as previously reported [51, 62]. The PCS2+ plasmids containing *prpf4* and *slc25a39* CDSs were linearized with HindIII, and were used as templates for in vitro transcription. DIG-labeled antisense RNA probes were synthesized using the T7 RNA polymerase and DIG RNA Labeling Mix (Roche) according to the manufacturer’s protocol.

### O-dianisidine staining

To examine the hemoglobin expression, zebrafish embryos were stained with o-dianisidine staining solution (Aladdin, 119-90-4) for approximately 45 min in the dark. After staining, embryos were rinsed three times with PBST (10 min each) and then fixed in 4% paraformaldehyde (PFA). Samples were stored at 4 °C in the dark prior to imaging [17]. The intensity of o-dianisidine staining was quantified using ImageJ.

### Western blot analysis

Western blotting was performed as described previously [63]. To analyze the protein levels of P53, γH2AX, and DNA-RNA hybrids in embryos subjected to different treatments, approximately 50-100 embryos were collected from each group for protein extraction. The primary antibodies used in this study were as follows: anti-p53 (GeneTex, GTX128135, 1:1000), anti-γH2AX (GeneTex, GTX127340, 1:1000), anti-DNA-RNA Hybrid Antibody (S9.6) (Antibody System, RGK60001, 1:1000), anti-phospho-Chk1 (Ser345) (Cell Signaling Technology, Cat# 348, 1:1000), anti-phospho-Chk2 (Ser33/35) (Cell Signaling Technology, Cat# 2665, 1:1000), and anti-GAPDH (absin, abs830030, 1:1000). The secondary antibodies were Goat Anti-Mouse IgG H&L (HRP) (Abcam, ab205719, 1:5000) and Goat Anti-Rabbit IgG (HRP) (GeneTex, GTX213110-01, 1:5000).

### Immunostaining

Embryos were fixed overnight at 4 °C in 4% paraformaldehyde. After fixation, they were washed three times with PBS for 5 min each and then blocked with PBTN (4% BSA, 0.02% NaN₃, in PT) at 4 °C for 2 h. Primary antibodies, including anti-active caspase-3 (BD, 559565, 1:500) and anti-γH2AX (GeneTex, GTX127340, 1:200), were diluted in PBTN and applied to the embryos. The embryos were incubated with the primary antibodies on a shaker at 4 °C overnight. Following primary antibody incubation, the embryos were washed with PT (0.3% Triton-X-100 in 1× PBS) for at least 20 min per wash, repeating this step 8 times. The secondary antibody, goat anti-rabbit IgG H&L (Alexa Fluor® 488) (Abcam, ab150081, 1:500), was diluted in PBTN and applied to the embryos. The embryos were incubated with the secondary antibody at 4 °C overnight in the dark. Finally, the embryos were washed more than 8 times with PT (30 min per wash) and prepared for imaging.

### TUNEL staining

Embryos were dechorionated and fixed in 4% paraformaldehyde overnight at 4 °C. After fixation, they were washed three times with PBST (10 min each) and stored in 100% methanol overnight at −20 °C. The following day, embryos were rehydrated by washing three times with PBST. Cell apoptosis was then detected using the In Situ Cell Death Detection Kit, Fluorescein (Roche, 11684795910) according to the manufacturer’s instructions.

### Microscopy

Images of whole-mount in situ hybridized embryos (in 100% glycerol) were taken at room temperature using an Olympus SZX16 microscope. Live embryos of the transgenic line Tg(*gata1*:DsRed) were embedded in 1% low-melting-point agarose (LMP agarose), and images of red precursor cells in the CHT region were captured using a Nikon A1 confocal laser scanning microscope. To obtain apoptosis images of cells in the CHT region from caspase-3 immunostained and TUNEL-stained embryos, the embryos were correctly oriented and embedded in 1% LMP agarose, followed by imaging with a Nikon A1 confocal laser scanning microscope. Live embryos of the transgenic lines Tg(*gata1*:DsRed) and Tg(*lyz*:DsRed) were anesthetized and imaged with an Olympus SZX16 microscope to assess the number of granulocytes and red precursor cells.

### Statistical analysis

Data were analyzed using NovoExpress, ImageJ, and GraphPad Prism 8 for Windows (GraphPad Software). Welch’s t-test (two-tailed, unequal variance) was performed to determine the statistical significance of the differences. Quantitative data are presented as the mean ± SD values, and all experiments were performed using at least three independent biological replicates. The individuals in each group were selected randomly. The fish sample size is more than 9 individuals, and all the sample sizes are shown in the figures and figure legends. Because the investigator must know each embryo is *prpf4* mutant or not, so the investigator was not blinded to the group allocation during the experiment. Statistical significance is indicated as follows: “ns”, not significant; “*” *p* < 0.05; “**” *p* < 0.01; “***” *p* < 0.001; “****” *p* < 0.0001.

## Supplementary information

Supplementary Materials

Table S1

Table S2

Table S3

Table S4

Gel Supplementary

## Data Availability

All data supporting the findings of this study are available in the main text or the supplementary materials. The raw RNA-seq datasets have been deposited in the NCBI Sequence Read Archive (SRA) under the accession number PRJNA1253320 and can be accessed at https://www.ncbi.nlm.nih.gov/sra/PRJNA1253320. Additional data or information are available from the corresponding author upon reasonable request.
