# Supplementary material for: Prpf4 sequentially regulates the expansion and maturation of erythrocyte through distinct mechanisms
Source: Cell Death Discov. 2025 Dec 8;11:555. doi: 10.1038/s41420-025-02846-6 (PMC12686395; doi:10.1038/s41420-025-02846-6)
Supplement: Supplementary file 1 — Supplementary Materials [file 41420_2025_2846_MOESM1_ESM.docx]

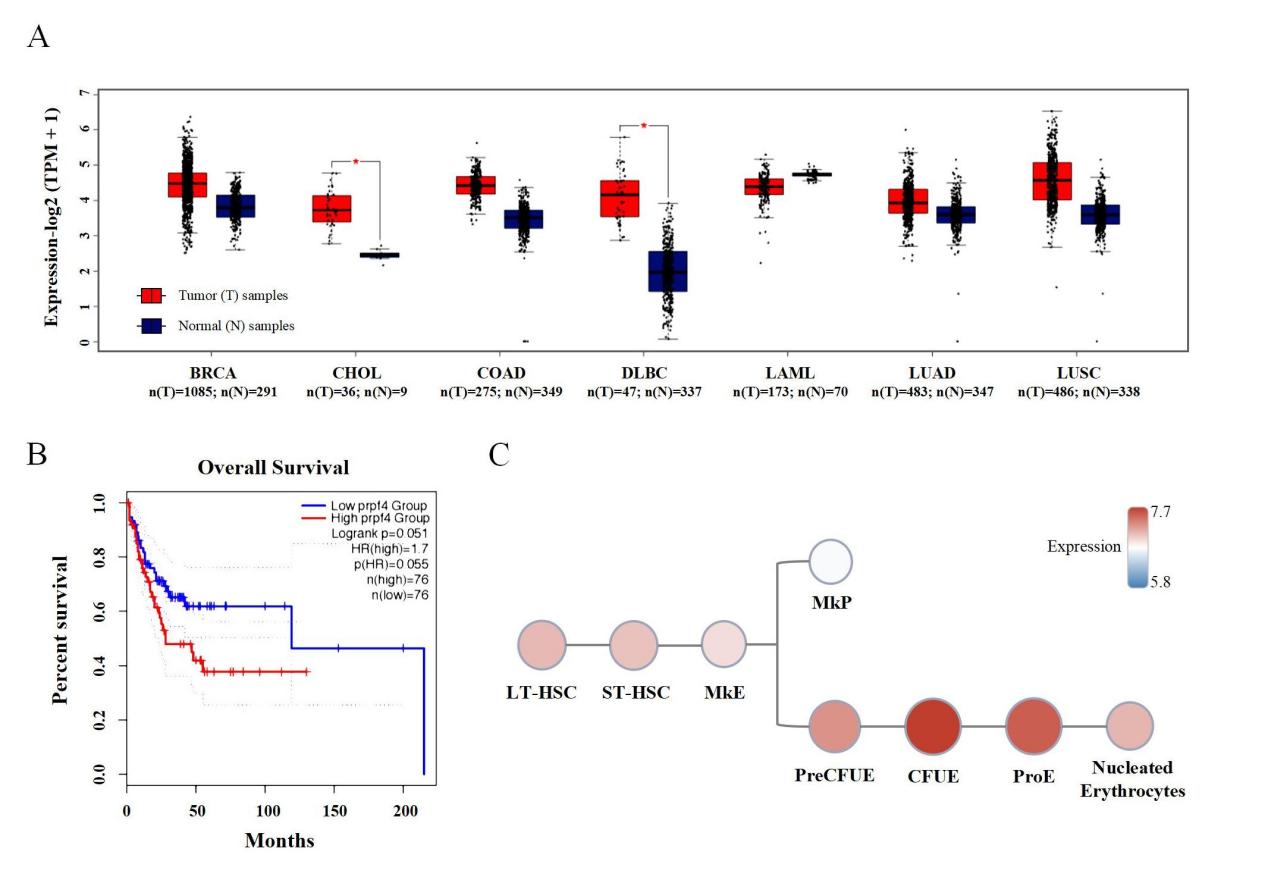


**Fig.S1 Expression pattern and clinical relevance of *PRPF4* in cancer and normal hematopoietic cells.**

**A** Expression levels of *PRPF4* in different cancer types. Box plots display the expression of *PRPF4* in tumor (red) and normal (blue) tissues across various cancers, including Breast Invasive Carcinoma (BRCA), Cholangiocarcinoma (CHOL), Colon Adenocarcinoma (COAD), Diffuse Large B-Cell Lymphoma (DLBC), Acute Myeloid Leukemia (LAML), Lung Adenocarcinoma (LUAD), and Lung Squamous Cell Carcinoma (LUSC). The number of tumor (T) and normal (N) samples is indicated below each box plot. Asterisks represent statistically significant differences. **B** Kaplan-Meier survival analysis of patients with diffuse large B-cell lymphoma (DLBC) stratified by *PRPF4* expression. Patients were divided into high (red) and low (blue) *PRPF4* expression groups. The overall survival rate was significantly lower in patients with high *PRPF4* expression compared to those with low *PRPF4* expression. **C** *Prpf4* mRNA expression in the normal mouse hematopoietic system was analyzed using the BloodSpot database. LT-HSC, Long term Hematopoietic stem cells; ST-HSC, Short term Hematopoietic stem cells; MkE, Megakaryocyte erythroid precursors; MkP, Megakaryocyte precursors; PreCFUE, Pre-colony-forming unit erythroid cells; CFUE, Colony-forming unit erythroid cells; ProE, Erythroid progenitor cells; Nucleated Erythrocytes, Nucleated Erythrocytes.


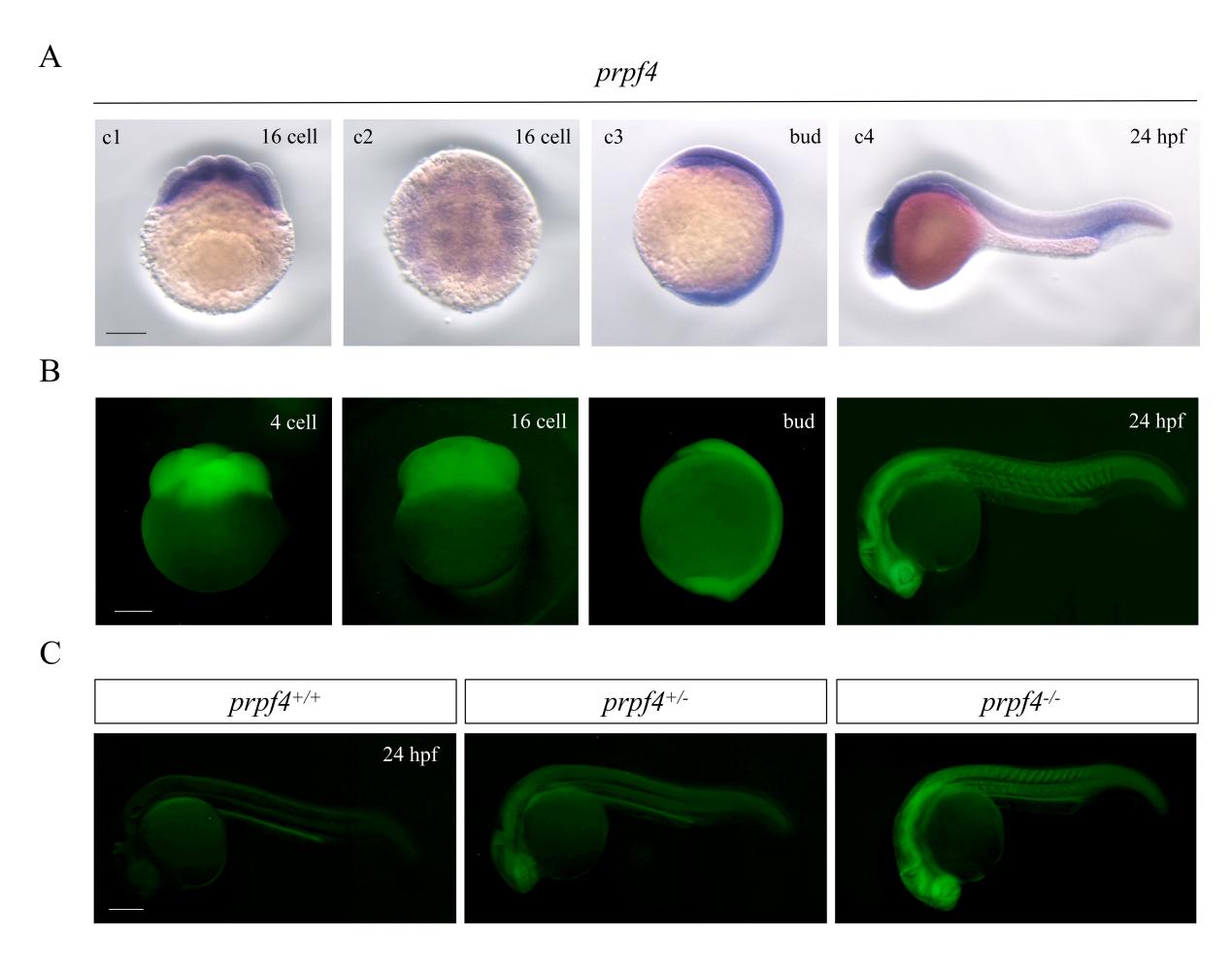


**Fig.S2 The expression pattern of *prpf4* in zebrafish embryos.**

**A** WISH of *prpf4* in WT embryos at the indicated stages shows that *prpf4* is maternally expressed and widely distributed throughout the embryo. **B** EGFP expression in *prpf4* mutant embryos at various developmental stages. **C** At 24 hpf, sibling wild-type (*prpf4*^+/+^) embryos show no EGFP fluorescence; heterozygous (prpf4^+/−^) embryos exhibit weak fluorescence; while homozygous mutant (prpf4^−/−^) embryos display strong EGFP fluorescence. Scale bar: 200 µm.


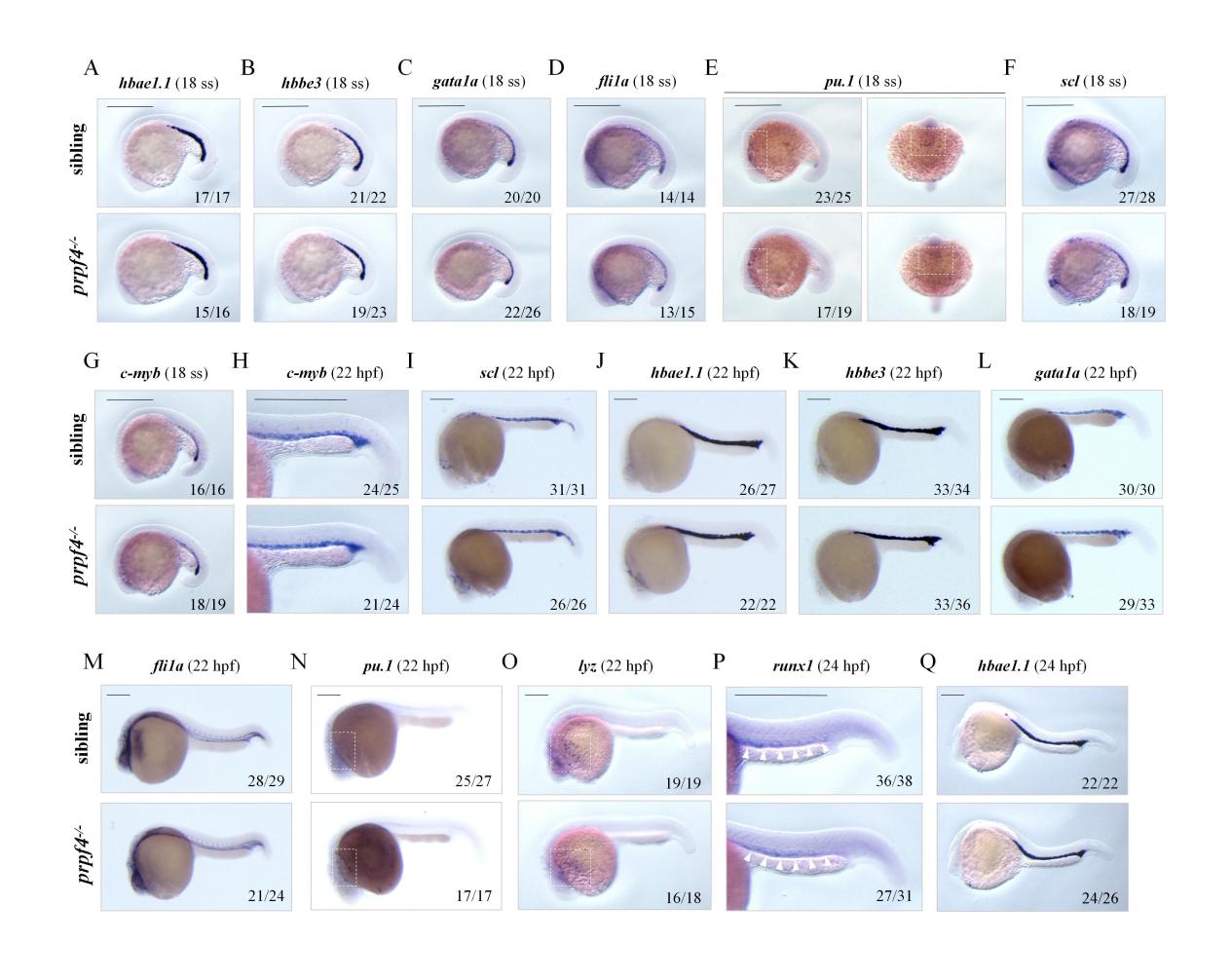


**Fig. S3 *prpf4* mutation does not affect primitive hematopoiesis in zebrafish embryos.**

**A-Q** WISH was performed to examine the expression of hematopoietic and lineage-specific markers in siblings and prpf4^−/−^ embryos at different developmental stages. At 18 somite stage : **(A)** *hbae1.1*, **(B)** *hbbe3*, **(C)** *gata1a*, **(D)** *fli1a*, **(E)** *pu.1* (dashed boxes indicate the *pu.1* expression domain), **(F)** *scl*, and **(G)** *c-myb*. At 22 hpf: **(H)** *c-myb*, **(I)** *scl*, **(J)** *hbae1.1*, **(K)** *hbbe3*, **(L)** *gata1a*, **(M)** *fli1a*, **(N)** *pu.1* (dashed boxes indicate the *pu.1* expression domain), and **(O)** *lyz*. At 24 hpf: **(P)** *runx1*(white arrowheads indicate expression along the dorsal aorta) and **(Q)** *hbae1.1*. Scale bars: 200 μm.


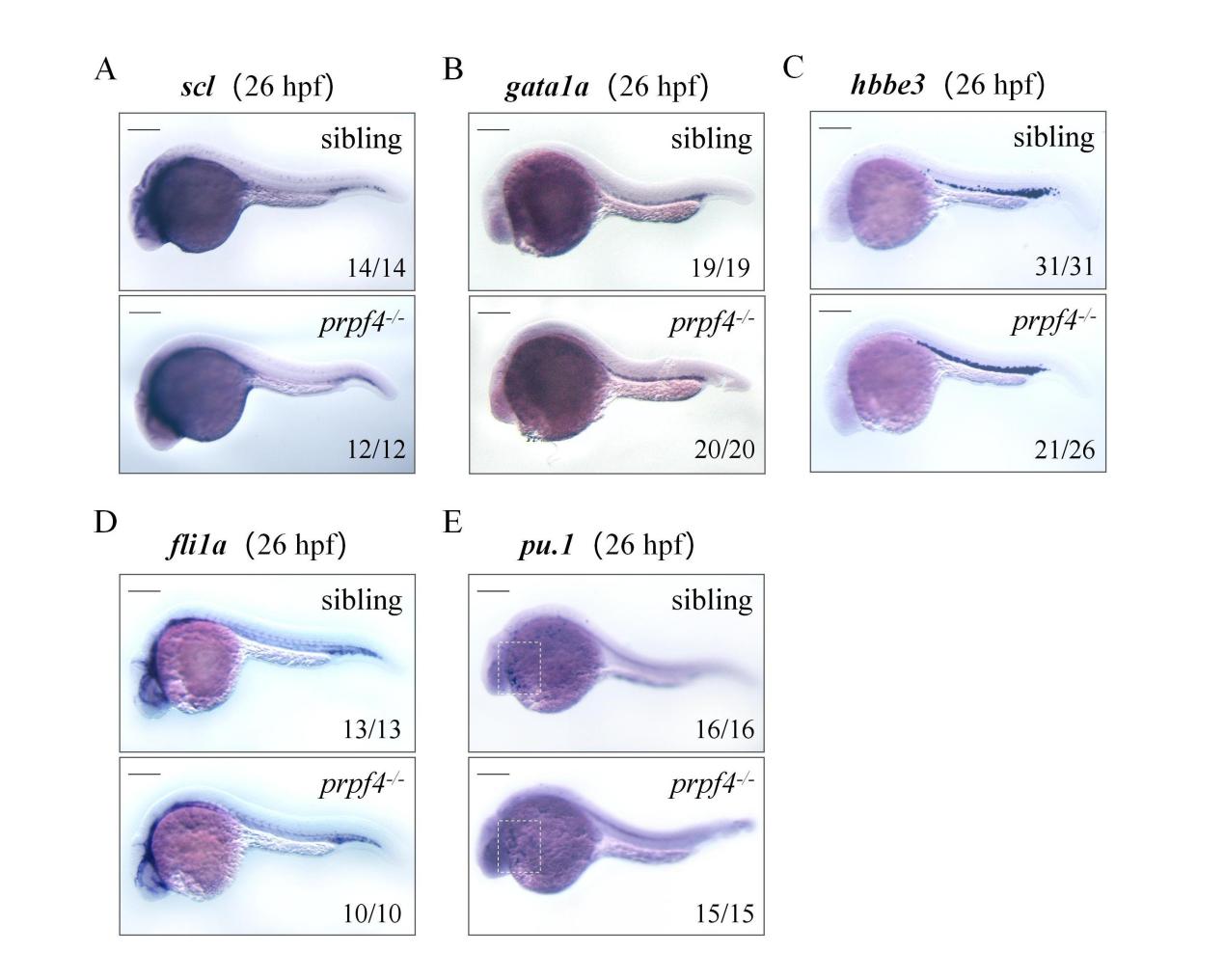


**Fig.S4 Expression of hematopoietic and endothelial marker genes in siblings and prpf4^−/−^ embryos.**

**A** WISH analysis of hematopoietic marker *scl* shows no significant differences between siblings and prpf4^−/−^ embryos*.* **B-C** Expression of *gata1a* and *hbbe3* at 26 hpf shows no significant differences between siblings and *prpf4*^−/−^ embryos. **D** Expression of *fli1a* at 26 hpf remains unaffected between prpf4^−/−^ embryos and siblings. **E** Expression of themyeloid marker *pu.1* shows no observable differences between siblings and prpf4^−/−^ embryos at 26 hpf. Scale bars: 200 μm.

**
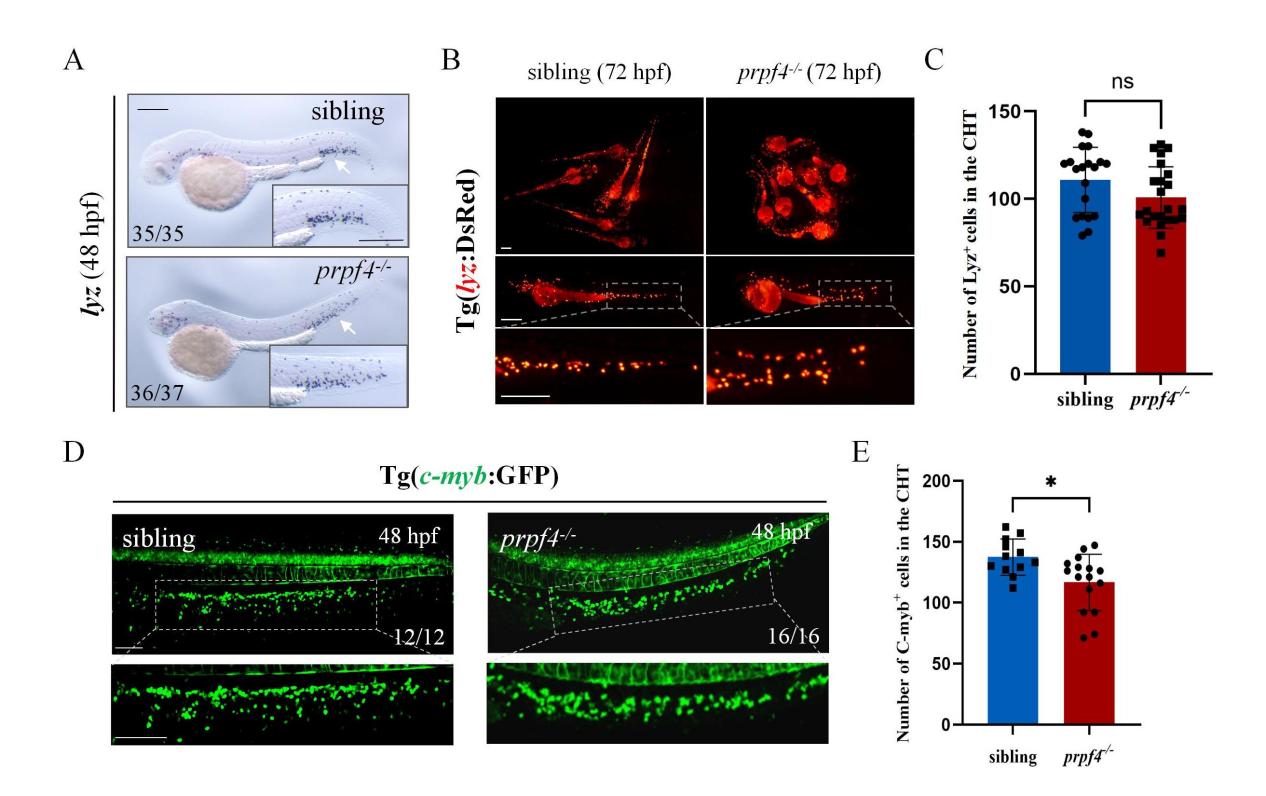
**

**Fig.S5 Effects of *prpf4* loss on myeloid cells and hematopoietic progenitor cells.**

**A** WISH analysis shows no difference in lyz expression between prpf4^−/−^ embryos and siblings at 48 hpf. **B-C** Distribution and quantification of neutrophils in siblings and prpf4^−/−^ embryos at 72 hpf using the Tg(*lyz*:DsRed) transgenic reporter line. **B** Distribution of Lyz^+^ cells in siblings and *p*rpf4^−/−^ embryos. Scale bar: 200 µm. **C** Quantification of Lyz^+^ cells in siblings and prpf4^−/−^ embryos from (**B**). siblings: n=20；prpf4^−/−^: n=23. **D** Confocal images of Tg(*c-myb*:GFP) embryos at 48 hpf showing C-myb⁺ HSPCs in the CHT. **E** Quantification of C-myb⁺ HSPCs corresponding to the images in **(D)**. Scale bars: 200 μm in **A–B**; 100 μm in **D**.


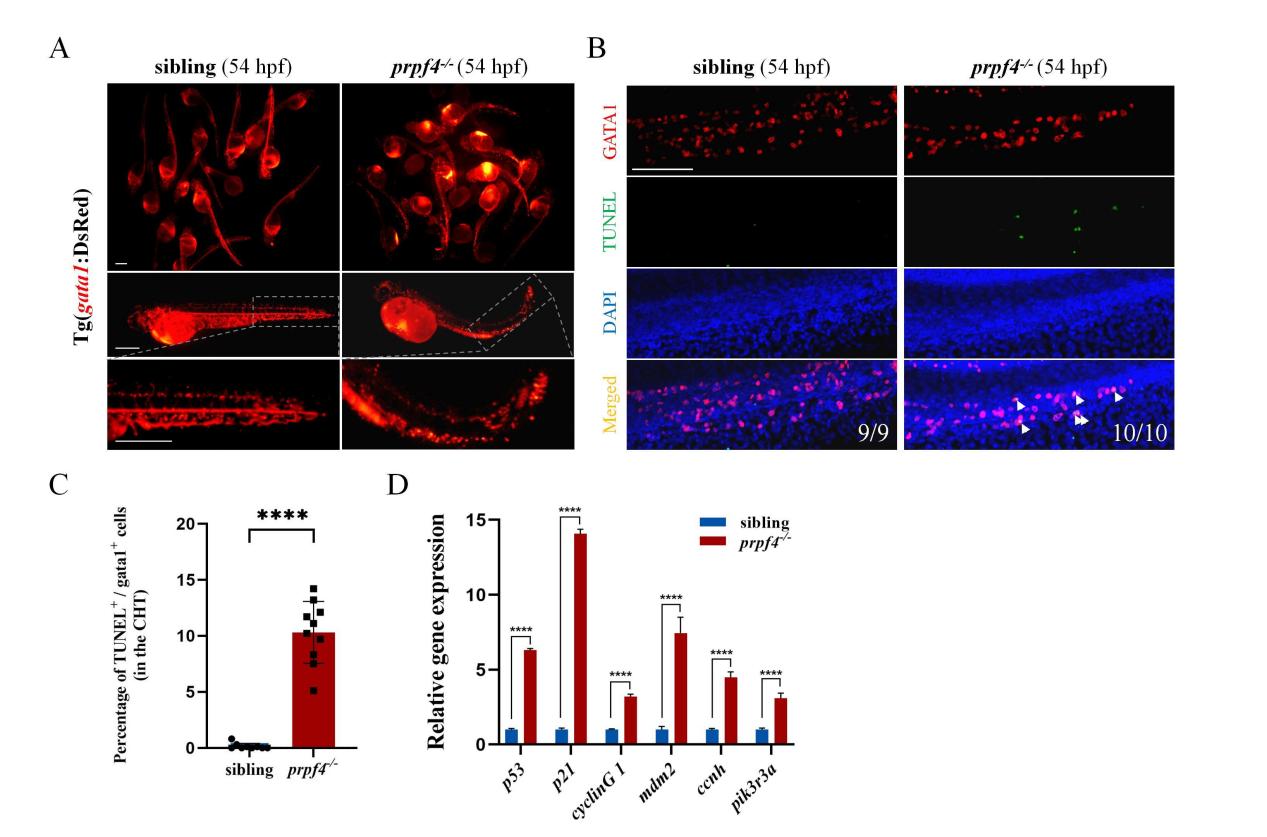
 **Fig.S6 Disruption of *prpf4* leads to hematopoietic defects, increased apoptosis, and altered gene expression in zebrafish.**

**A** Distribution of GATA1^+^ cells in siblings and prpf4^−/−^ embryos. Scale bar: 200 µm. **B-C** Confocal microscopy analysis of apoptosis in GATA1^+^ cells in the CHT region of siblings and prpf4^−/−^ embryos at 54 hpf (siblings: n = 9, prpf4^−/−^: n = 10). **B** TUNEL staining showing GATA1 (red), TUNEL (green), and DAPI (blue). Arrows indicate apoptotic GATA1^+^ cells. Scale bar: 100 µm. **C** Quantitative analysis shows a significantly higher proportion of apoptotic GATA1^+^ cells in the CHT region of prpf4^−/−^ embryos compared with siblings. **D** Elevated expression of cell cycle and DNA damage-related genes in prpf4^−/−^ mutants and siblings.


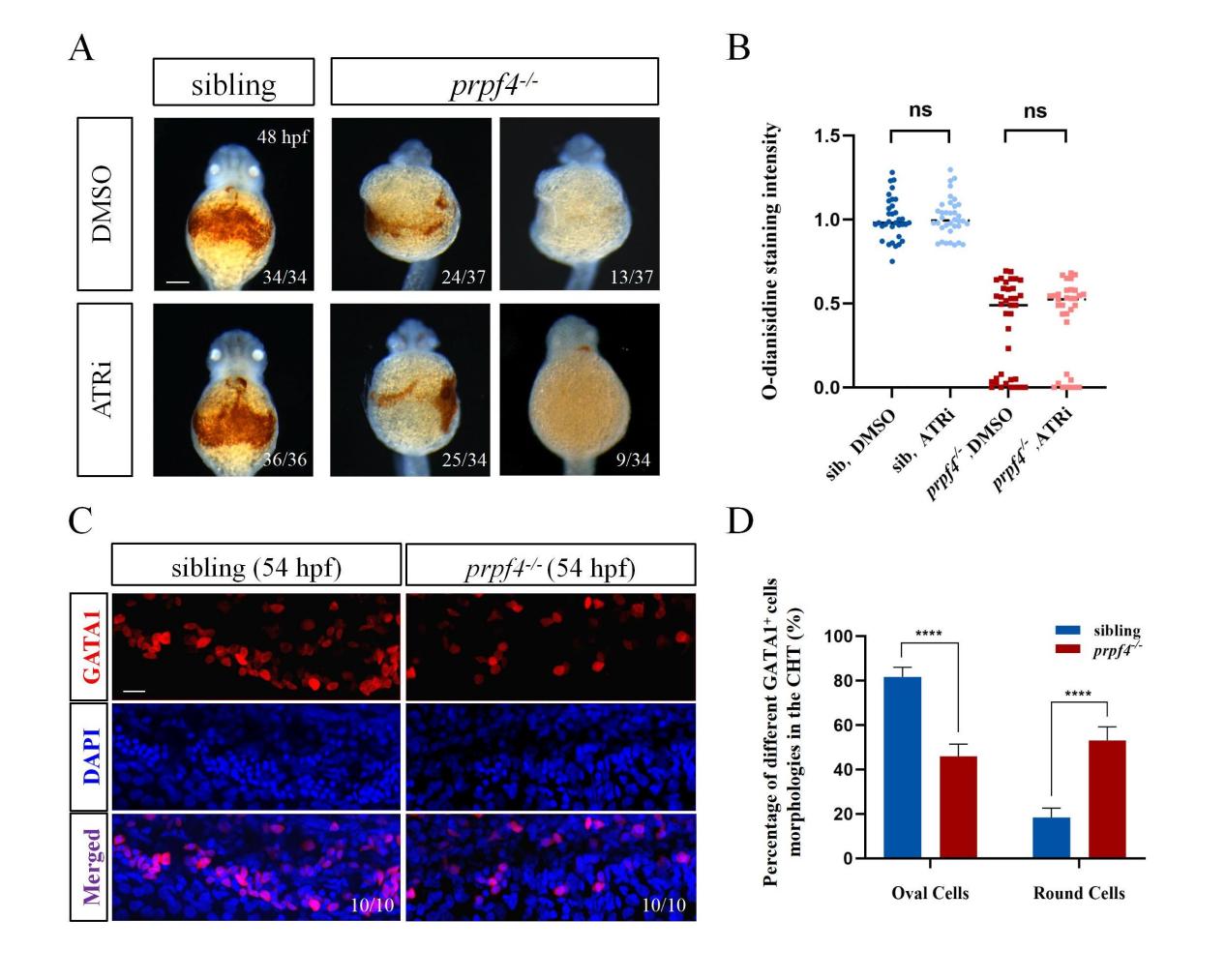


**Fig.S7 ATR inhibition fails to rescue anemia in *prpf4* mutants, and *prpf4* mutation impedes erythrocyte maturation.**

**A** O-dianisidine staining showing hemoglobin distribution in siblings and prpf4^−/−^ embryos at 48 hpf in the presence of DMSO or ATR inhibitor (ATRi). Scale bar: 200 µm. **B** Quantification of o-dianisidine staining intensity from **(A)**. **C** Morphological analysis of GATA1^+^ erythrocytes in the CHT region at 54 hpf using the Tg(*gata1*:DsRed) transgenic reporter. The morphology and distribution of GATA1^+^ erythrocytes are shown in siblings and prpf4^−/−^ embryos are shown. Scale bar: 20 µm. **D** Quantification of the percentage of GATA1^+^ erythrocytes with different morphologies in the CHT region. Siblings exhibit a significantly higher proportion of oval erythrocytes, while prpf4^−/−^ embryos show a significantly increased proportion of round erythrocytes .


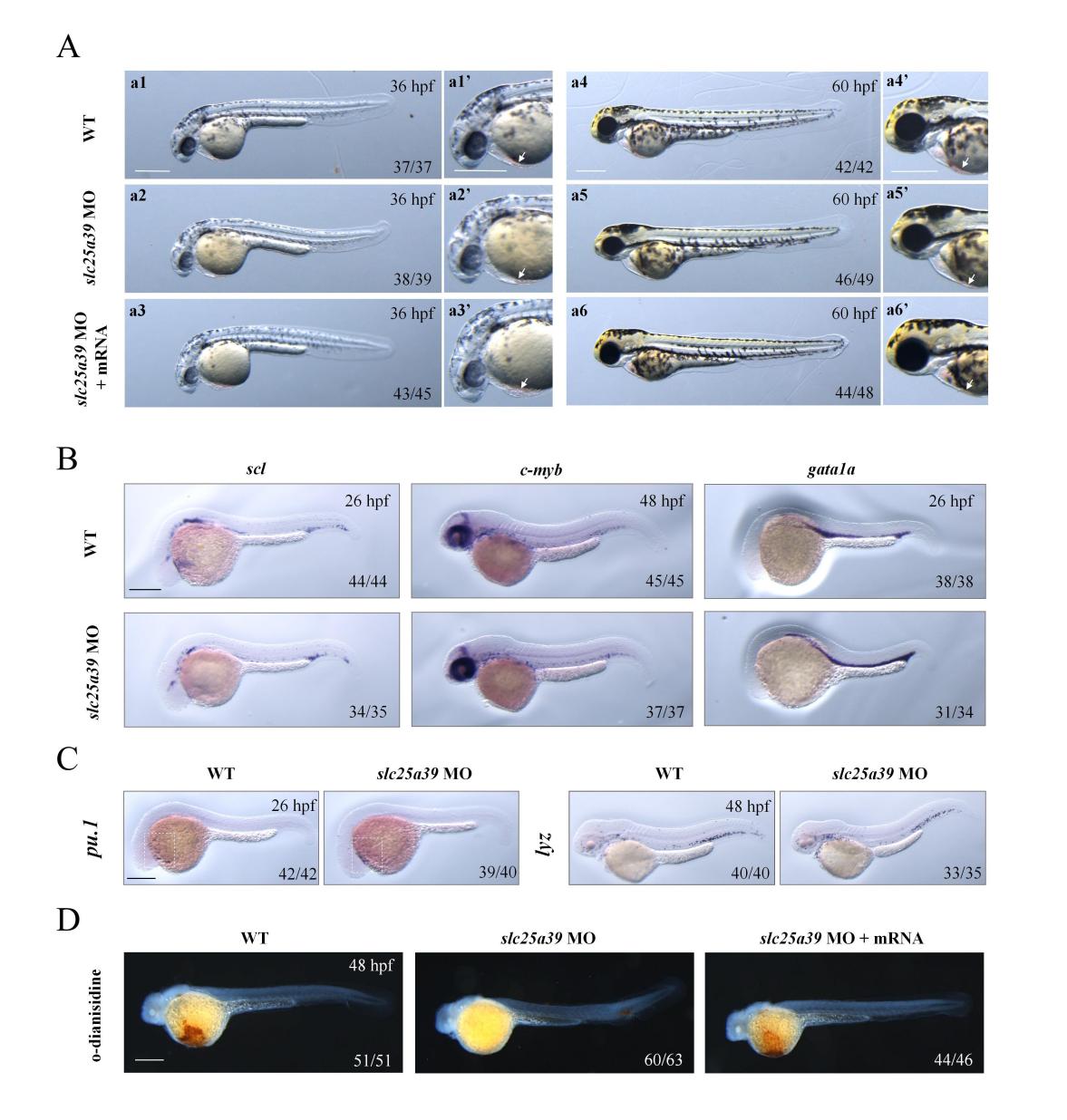


**Fig.S8 Knockdown of *slc25a39* leads to hemoglobin defects.**

**A** Embryos with *slc25a39* deficiency exhibit abnormal blood coloration. At both 36 hpf and 60 hpf, *slc25a39* morphants show normal overall morphology without obvious malformations but exhibited pronounced lightening of blood coloration in the precardiac and cardiac regions (**a1**–**a2’**, **a4**–**a5’**). Co-injection of *slc25a39* mRNA partially rescued the lightened blood coloration in *slc25a39* morphants (**a3**, **a6**). White arrows indicate the ventral yolk sac region in 36 hpf embryos (**a1’**–**a3’**) and the cardiac region in 60 hpf embryos (**a4’**–**a6’**). **B** Expression patterns of hematopoietic marker genes in *slc25a39* morphants and WT embryos at 26 hpf. Wild-type and *slc25a39* morphants exhibit comparable expression levels of *scl*, *c-myb*, and *gata1a*. **C** Expression patterns of *pu.1,* and *lyz* in wild-type embryos and *slc25a39* morphants. The dashed squares highlight the region of *pu.1* expression in the anterior region. **D** Knockdown of *slc25a39* results in hemoglobin production defects in zebrafish embryos. O-dianisidine staining shows significantly reduced hemoglobin levels in *slc25a39* morphants compared with WT, while co-injection of *slc25a39* MO and *slc25a39* mRNA partially rescues the hemoglobin defect in *slc25a39* morphants. Scale bar: 200 µm.


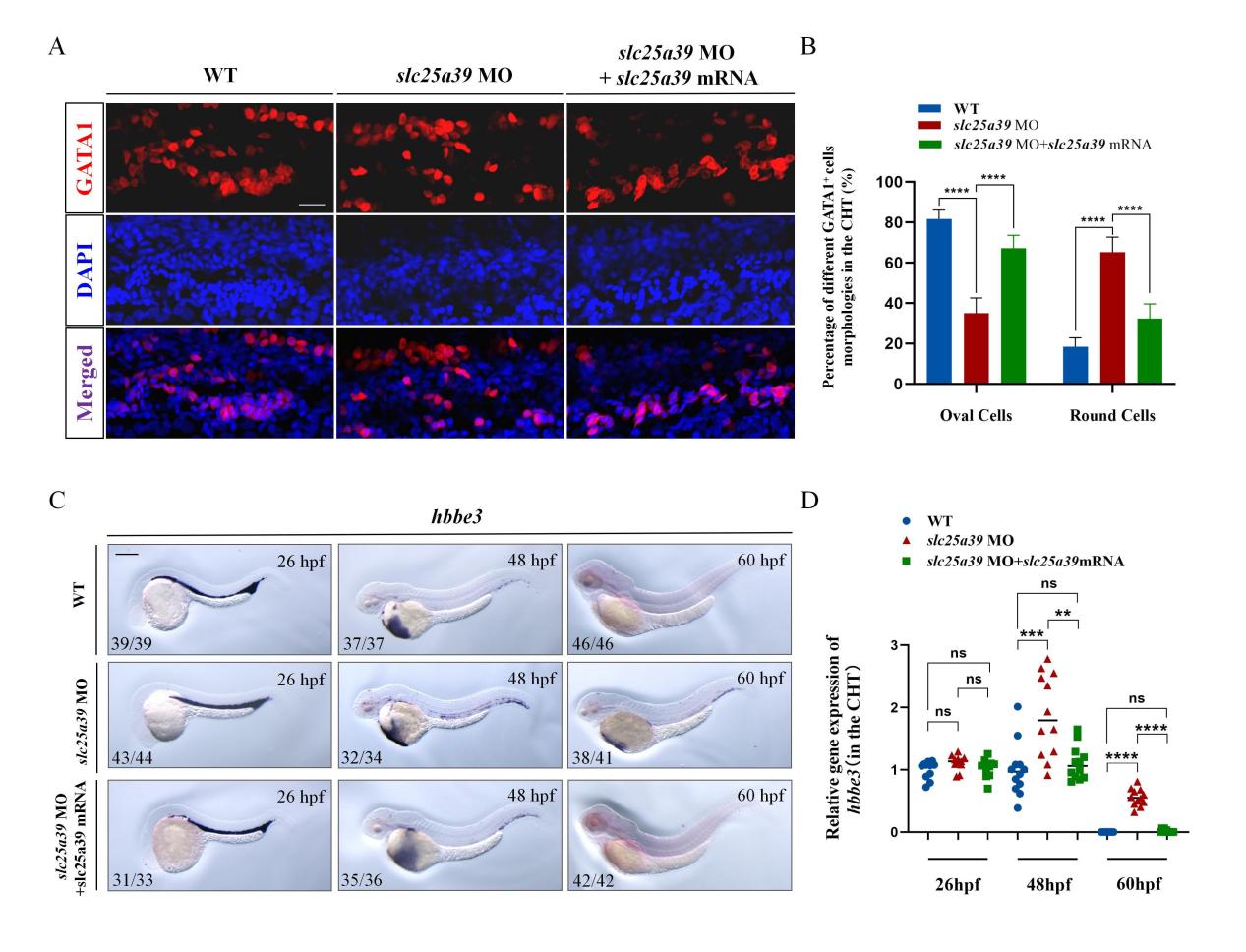


**Fig.S9 Knockdown of *slc25a39* impairs erythrocyte maturation.**

**A** Transgenic zebrafish embryos expressing Tg(*gata1*:DsRed) were used to visualize cell morphology in the CHT region of WT, *slc25a39* morphants, and the *slc25a39* MO + *slc25a39* mRNA co-injection group (n=11 embryos per group). Scale bar: 20 μm. **B** Quantification of the percentage of GATA1⁺ cells with different morphologies in the CHT region. Knockdown of *slc25a39* results in a significant increase in round cells, whereas co-injection of *slc25a39* mRNA significantly reduces the number of round cells and restores oval-shaped cells. **C** WISH analysis of *hbbe3* expression in WT, *slc25a39* morphants, and *slc25a39* morphants co-injected with *slc25a39* mRNA at different developmental stages (26 hpf, 48 hpf, and 60 hpf). Scale bar: 200 µm. **D** Quantification of *hbbe3* WISH staining intensity in the CHT at 26 hpf, 48 hpf, and 60 hpf.
