# Supplementary material for: Prpf4 sequentially regulates the expansion and maturation of erythrocyte through distinct mechanisms
Source: Cell Death Discov. 2025 Dec 8;11:555. doi: 10.1038/s41420-025-02846-6 (PMC12686395; doi:10.1038/s41420-025-02846-6)
Supplement: Supplementary file 2 — Table S1 [file 41420_2025_2846_MOESM2_ESM.docx]

| **Plasmid Construction**  **Table S1: Primer Sequences** | |
| --- | --- |
| PCS^2+^_F | 5’-CTCGAGCCTCTAGAACTATAGTG-3’ |
| PCS^2+^_R | 5’-TGGTGTTTTCAAAGCAACGATATCG-3’ |
| *slc25a39*_F | 5’-GTTGCTTTGAAAACACCAGCAGGATGGGAGATAGGCCTG-3’ |
| *slc25a39*_R | 5’-AGTTCTAGAGGCTCGAGCAGCTCAAAGTCCACACCTCGC-3’ |
| *prpf4*_F | 5’-GTTGCTTTGAAAACACCAGTCGTGTGGTCGTGTTTGTGG-3’ |
| *prpf4*_R | 5’-AGTTCTAGAGGCTCGAGCTGACATCCAGAGTTTAAAGG-3’ |
| **RT-qPCR** | |
| *beta-actin*_F | 5’-GCTGTTTTCCCCTCCATTGTT-3’ |
| *beta-actin*_R | 5’-TCCCATGCCAACCATCACT-3’ |
| *abcb7*_F | 5’-ACCTGAGTGATGCGCCAAACA-3’ |
| *abcb7*_R | 5’-GAAGCCCAGATCCAGGTTATG-3’ |
| *abcb10*_F | 5’-TACGTGTCACCCAGTTTGGCA-3’ |
| *abcb10*_R | 5’-CTGCACCTCTGTCAGCTCTTT-3’ |
| *uros*_F | 5’-GACTACTTCACTCAGCAGGGT-3’ |
| *uros*_R | 5’-GTCAATCCACGAGCCTGCAGA-3’ |
| *slc25a39*_F | 5’-GCCATAGCAGCAGAATCAAGACC-3’ |
| *slc25a39*_R | 5’-CTCGTTGTGGGTTATTTTTACAAATGCATC-3’ |
| ***ae1***: *hbae1.1*_F | 5’-CCAGGATGTTGATTGTCTAC-3’ |
| ***ae1***: *hbae1.1*_R | 5’-CAGTCTTGCCGTGTTTC-3’ |
| ***ae3***: *hbae3*_F | 5’-CCTAAGCCCCAACTCTC-3’ |
| ***ae3***: *hbae3*_R | 5’-CTCCCTTCAGGTCATCC-3’ |
| ***βe1***: *hbbe1.1*_F | 5’-CTTGACCATCGTTGTTG-3’ |
| ***βe1***: *hbbe1.1*_R | 5’-GATGAATTTCTGGAAAGC-3’ |
| ***βe3***: *hbbe3*_F | 5’-ATGCTTGGTCGTCTATCC-3’ |
| ***βe3***: *hbbe3*_R | 5’-ATGATTGCCTCTGTGTTG-3’ |
| ***ae5***: *hbae5*_F | 5’-CCTCAGACCAAGACCTAC-3’ |
| ***ae5***: *hbae5*_R | 5’-TCAGACAGAGCCAAAGC-3’ |
| ***βe2***: *hbbe2*_F | 5’-ACTATGAGGAGGCTGGAC-3’ |
| ***βe2***: *hbbe2*_R | 5’-CGGCGTAGGTGTTCTTG-3’ |
| ***p53***_F | 5’-TTGCCGGGATCGTTTGACC-3’ |
| ***p53***_R | 5’-ATAGATGGCAGTGGCTCGAA-3’ |
| ***p21***_F | 5’-TCAGTCATGACAGCTCAGAGGCGCAGA-3’ |
| ***p21***_R | 5’-GTCGCGATGCGTCCTCCAGATC-3’ |
| ***cyclinG1*_F** | 5’-GCTTTAAACTTACATACTGAGA-3’ |
| ***cyclinG1*_R** | 5’-ACGCGGAGTCCACAGAGTTTG-3’ |
| ***mdm2*_F** | 5’-TTGAAAAGCCTGTTAAG-3’ |
| ***mdm2*_R** | 5’-CACTCCAAGAACAGCAACG-3’ |
| ***ccnh*_F** | 5’-TGTCCTCAATAGCGCTGCAC-3’ |
| ***ccnh*_R** | 5’-TGGGAAGCTCGTACGCTTTA-3’ |
| ***pik3r3a*_F** | 5’-ATTGCTACGCCTGCTCTGTC-3’ |
| ***pik3r3a*_R** | 5’-GATTTGTGGAGGTCGAAGGGT-3’ |
| **SqRT-PCR** | |
| *rps27l*_F | 5’-TCAACCCGTCCTTTGATTTGG-3’ |
| *rps27l*_R | 5’-ATGTAAGGCATCAACAAGTCAC-3’ |
| *abcb7*_F | 5’-ACGGGTTGCTCTTTCAGCTC-3’ |
| *abcb7*_R | 5’-CCTGCTGTGGTTCATAGAATCGAAA-3’ |
| csnk1da_F | 5’-GAGCCCCTGTCAACGTTT-3’ |
| csnk1da_R | 5’-CCTCCCATCTTGTCAGCGATT-3’ |
| *haus6*_F | 5’-GATGTCCTTGTGCCCTCTGA-3’ |
| *haus6*_R | 5’-TTTCCTAGATGAAAAGGGATCCGA-3’ |
| *jak2b*_F | 5’-TATGAACTCTTTACATACAGCGACAA-3’ |
| *jak2b*_R | 5’-CTTTCTTTAAAACTCAGGCACTTGGGTA-3’ |
| *smarca1*_F | 5’-TGTGTACGAAATGCGCCTCA-3’ |
| *smarca1*_R | 5’-ACAAGTGTTTAATACGTCCCAA-3’ |
| *slc25a39*_F | 5’-CACACCTCTGGATGTAGTGA-3’ |
| *slc25a39*_R | 5’-CTCGTTGCGGGTTATTTTTACAAATGCATC-3’ |
| *slc25a37*_F | 5’-AGGGGCCTCAATATCACT-3’ |
| *slc25a37*_R | 5’-ATGAAATGAACGGCCTGGAA-3’ |
| *beta-actin*_F | 5’-GCTGTTTTCCCCTCCATTGTT-3’ |
| *beta-actin*_R | 5’-TCCCATGCCAACCATCACT-3’ |
| *hbae1.1*_F | 5’-CTCTCCAGGATGTTGATTGTC-3’ |
| *hbae1.1*_R | 5’-AGGAGTCCGGCATTAAGGTCA-3’ |
