# Supplementary material for: Prpf4 sequentially regulates the expansion and maturation of erythrocyte through distinct mechanisms
Source: Cell Death Discov. 2025 Dec 8;11:555. doi: 10.1038/s41420-025-02846-6 (PMC12686395; doi:10.1038/s41420-025-02846-6)
Supplement: Supplementary file 6 — Gel Supplementary [file 41420_2025_2846_MOESM6_ESM.docx]

**Fig. 3A Gel supplementary**


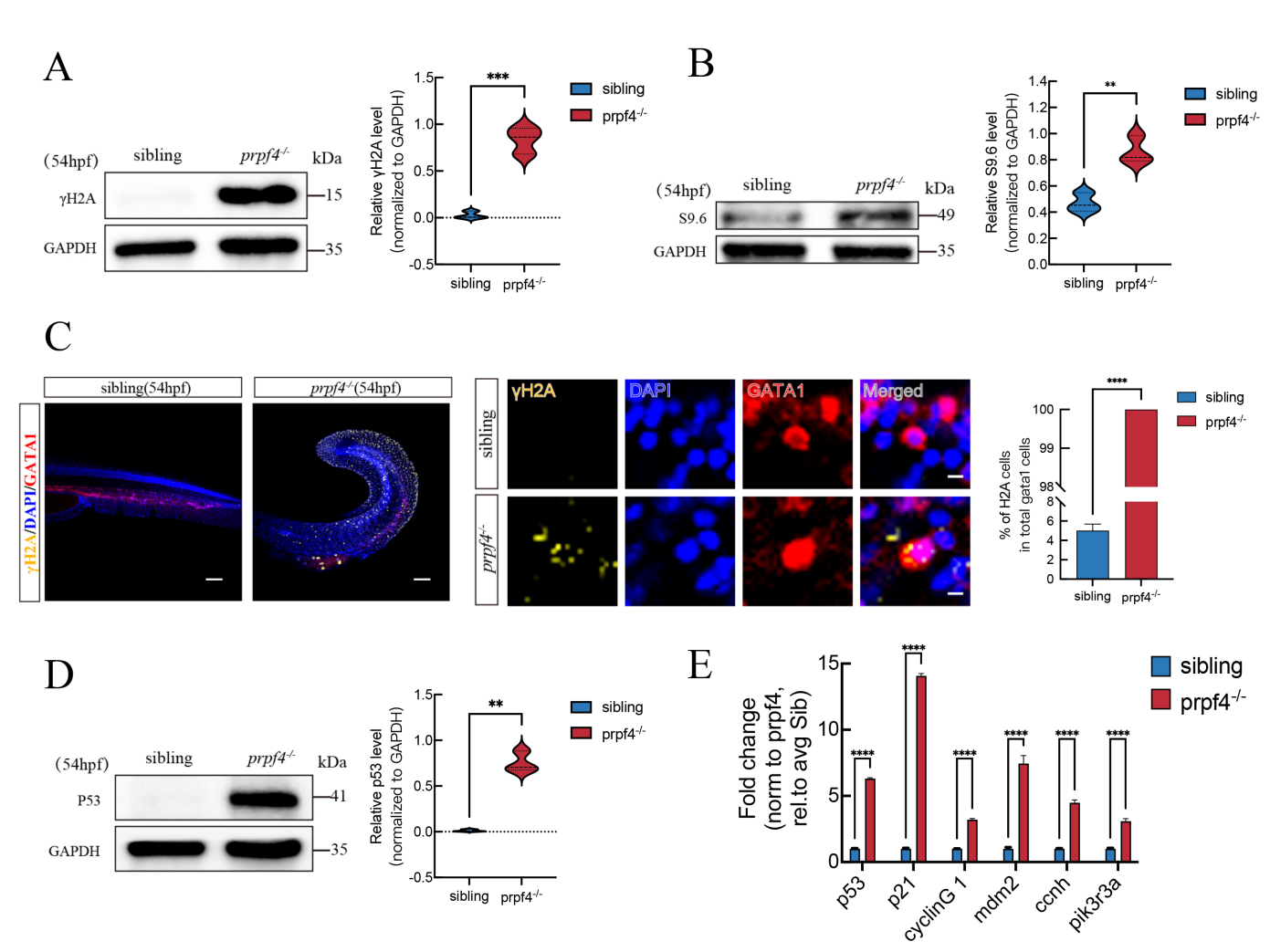


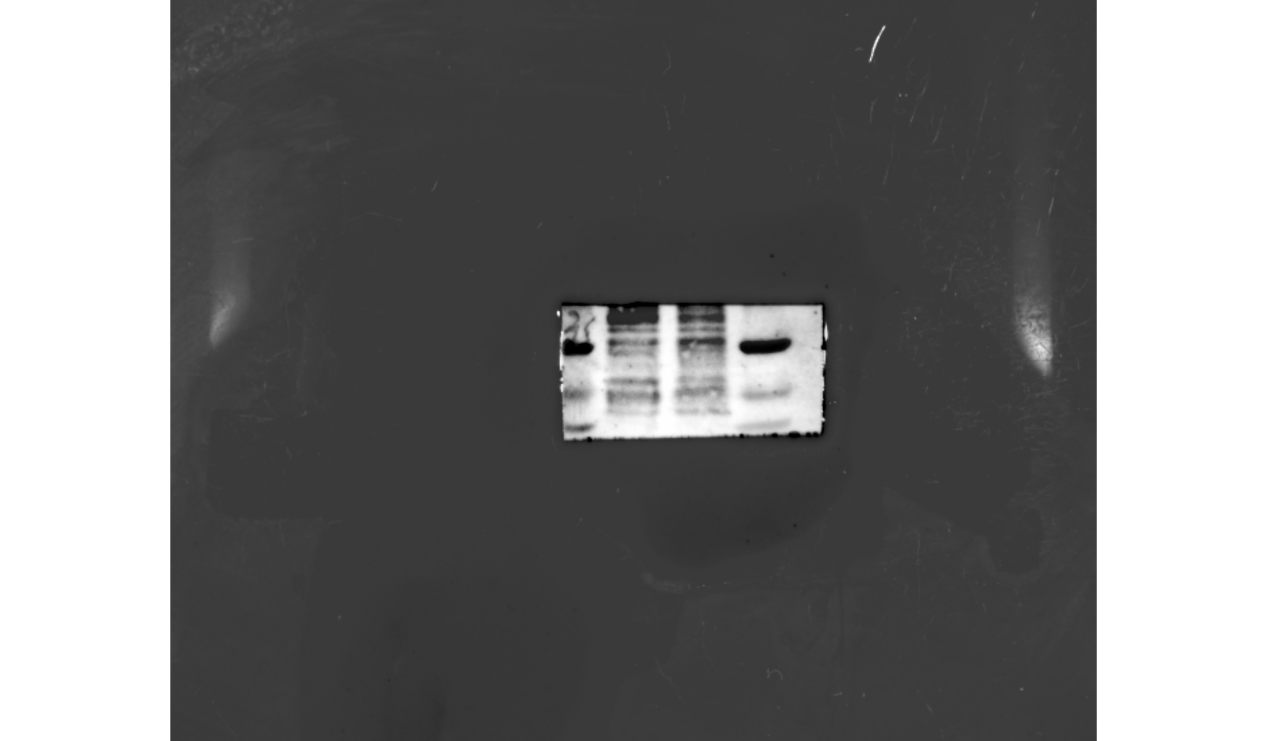

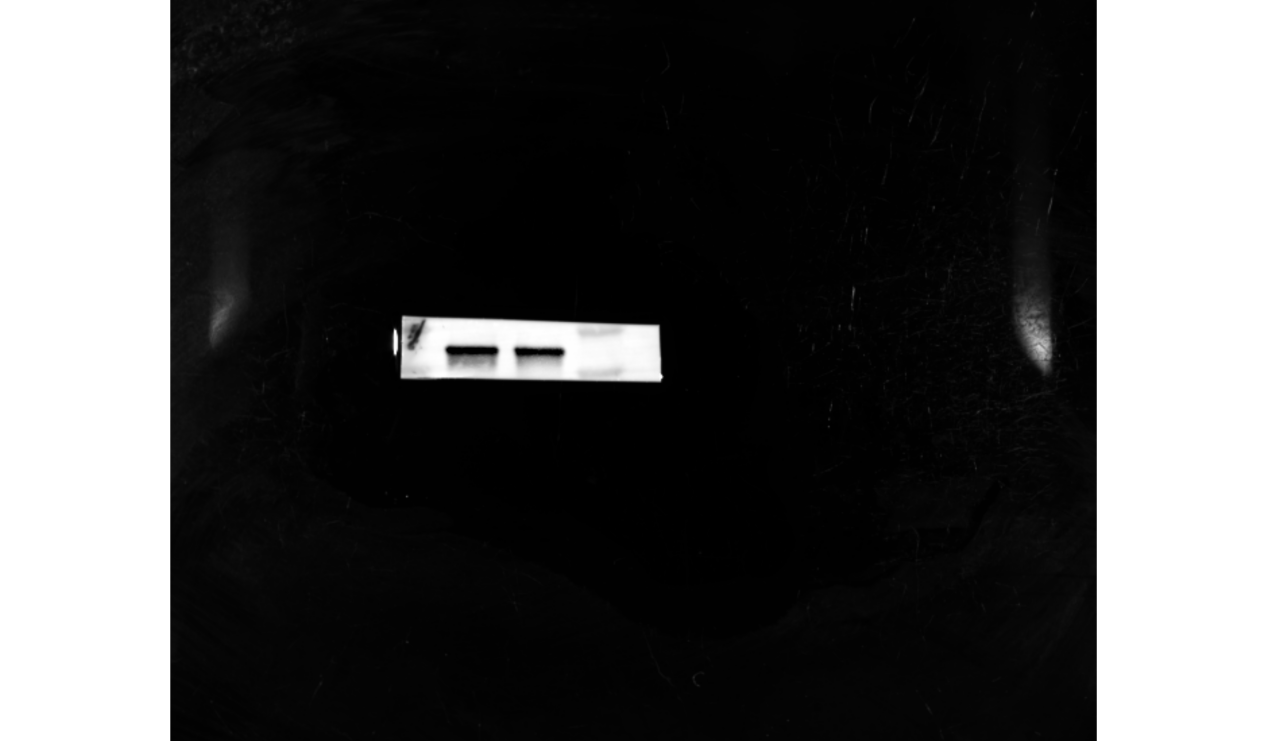


S9.6

GAPDH

**Fig. 3B Gel supplementary**


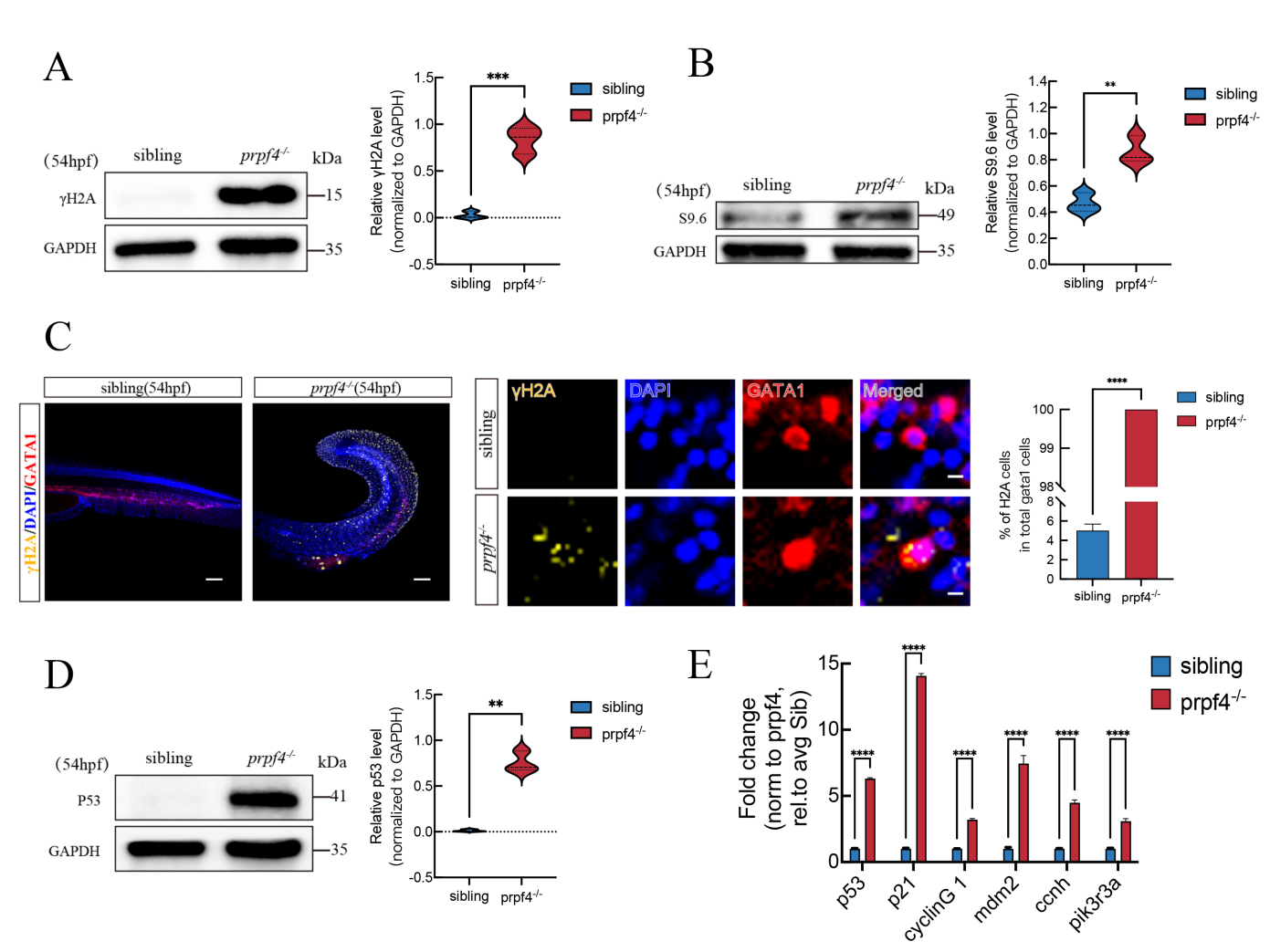

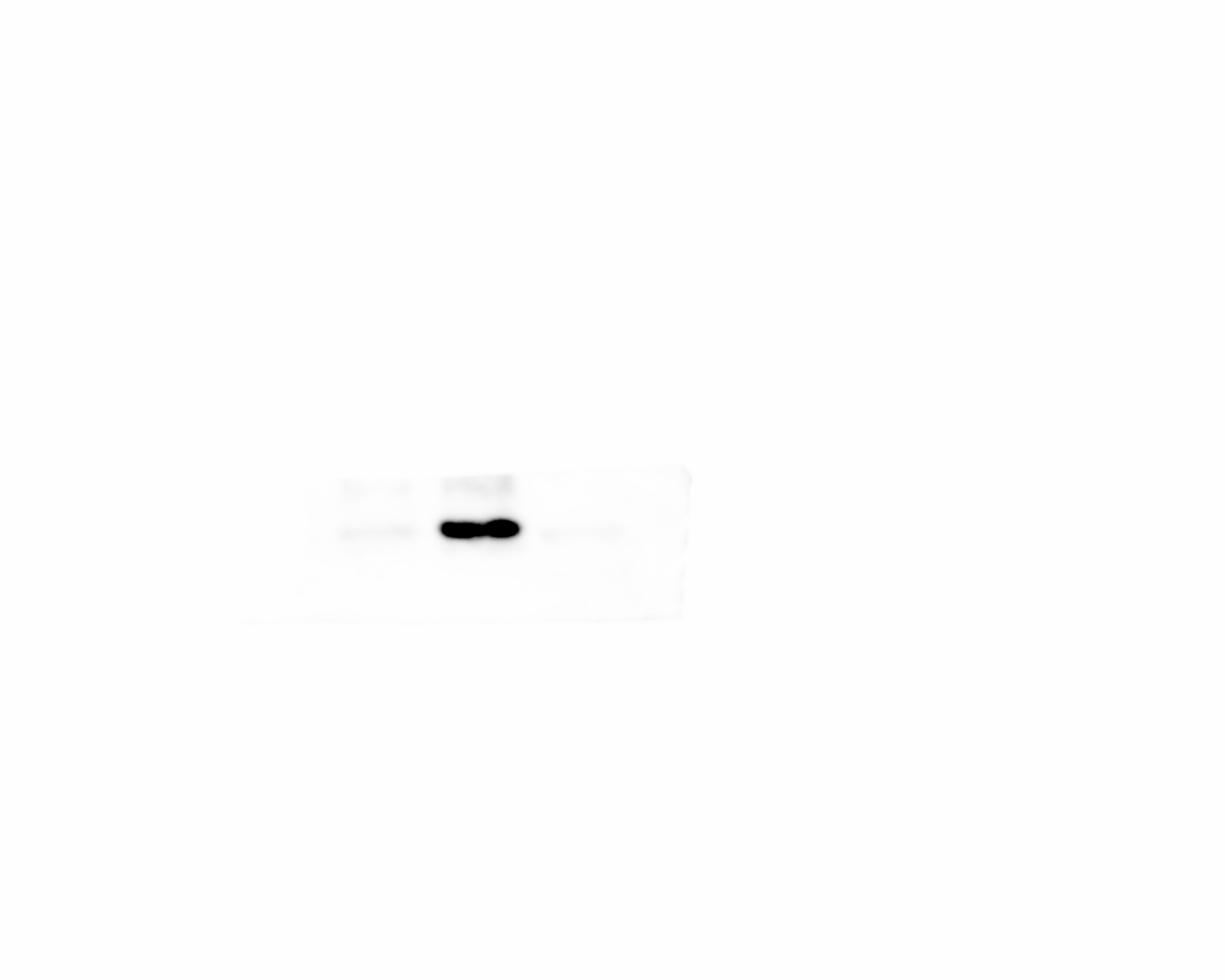


γH2A


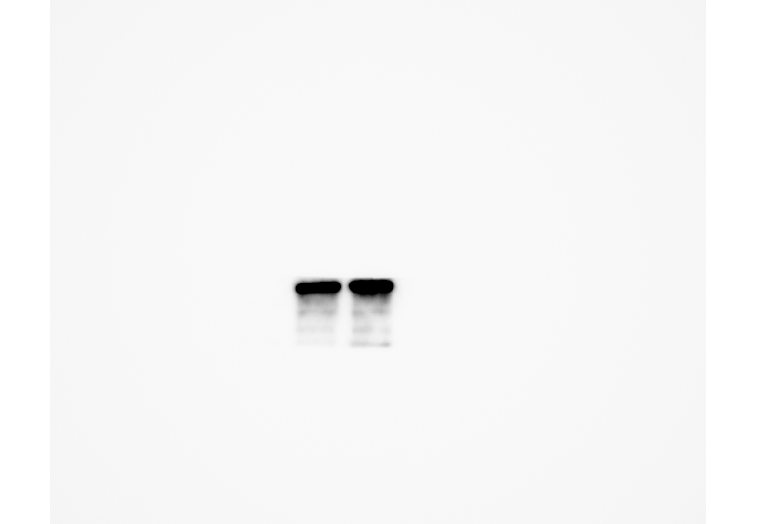


GAPDH

**Fig. 3F Gel supplementary**


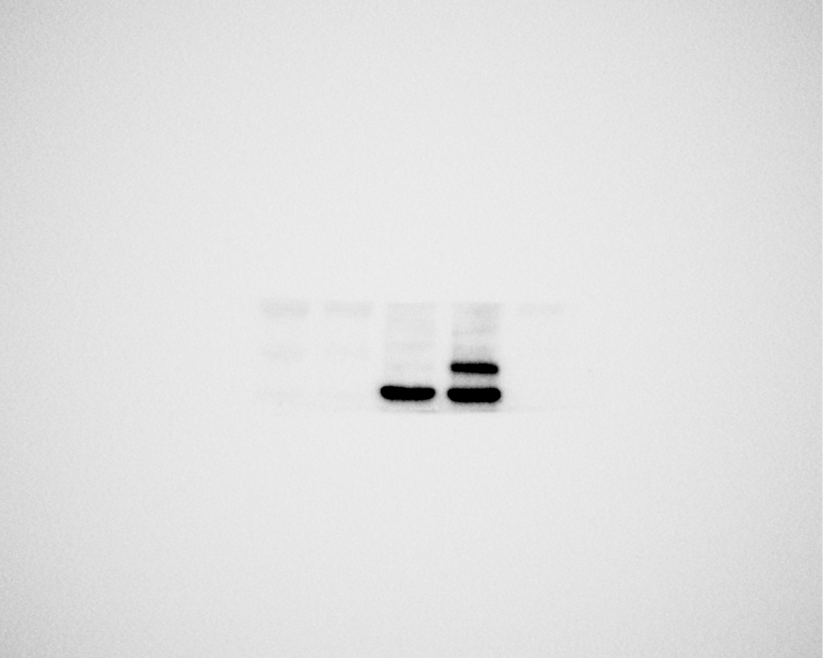


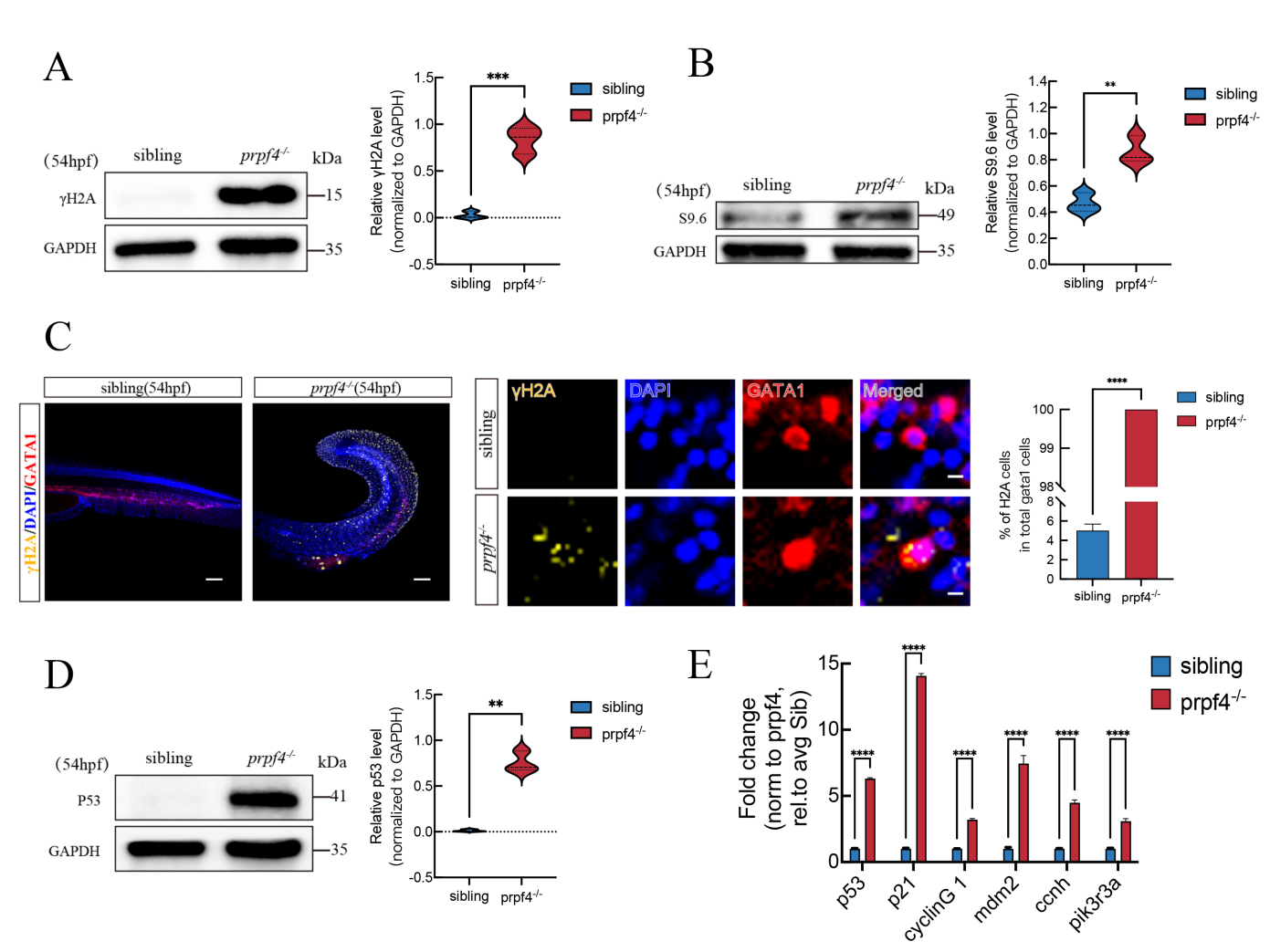


P53


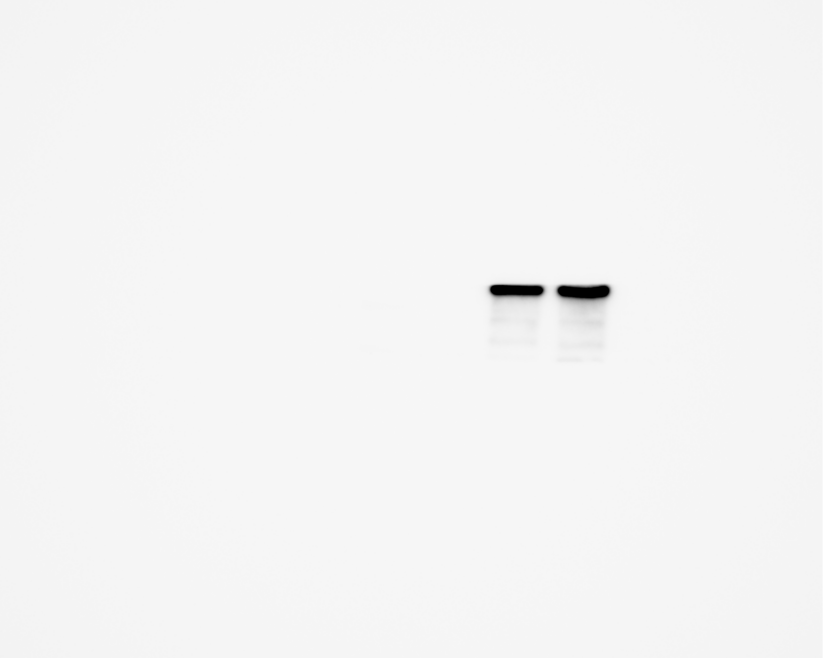


GAPDH

**Fig. 3G Gel supplementary**


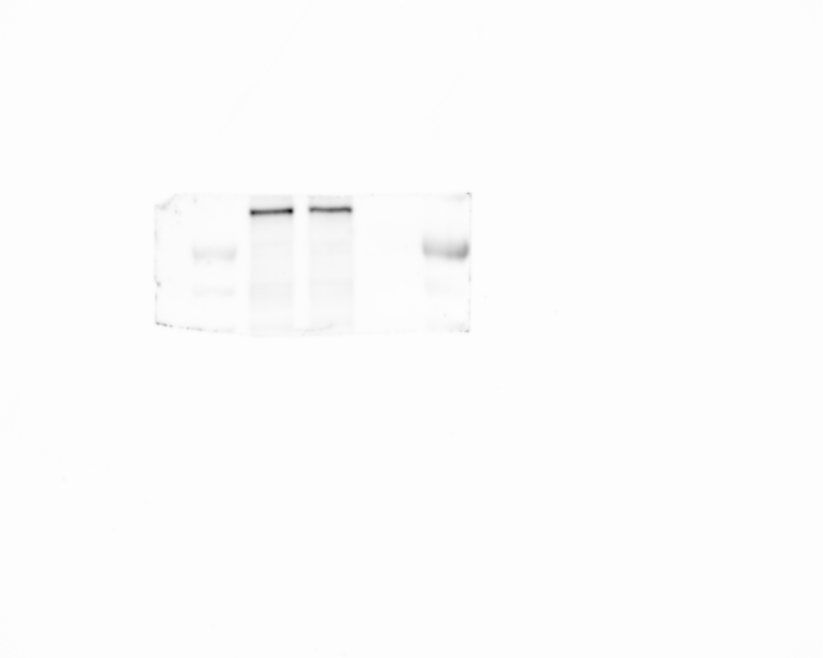


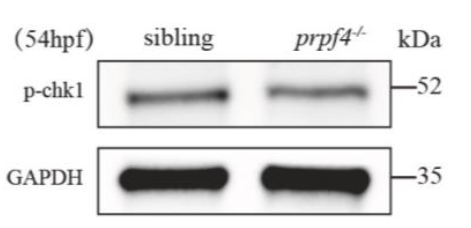


p-chk1

GAPDH


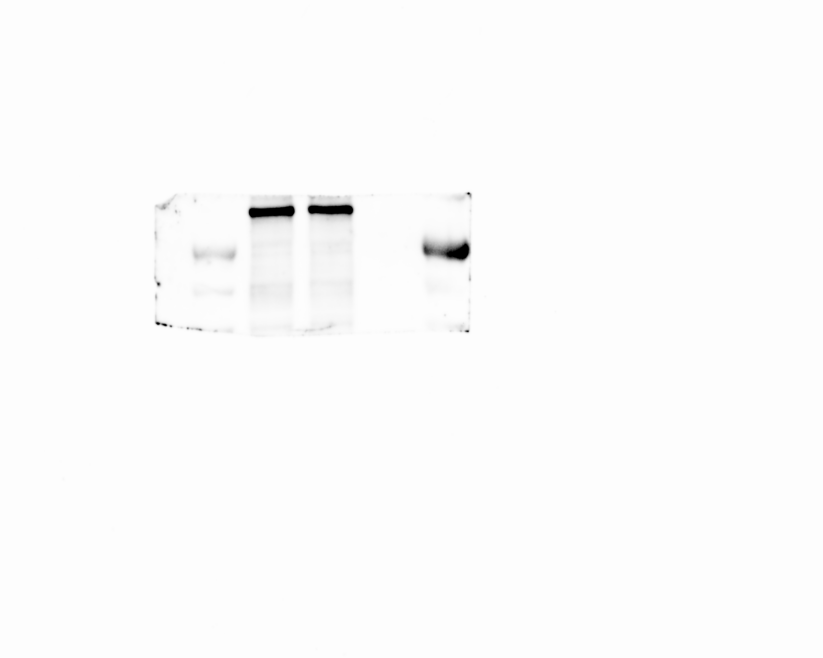


**Fig. 3H Gel supplementary**


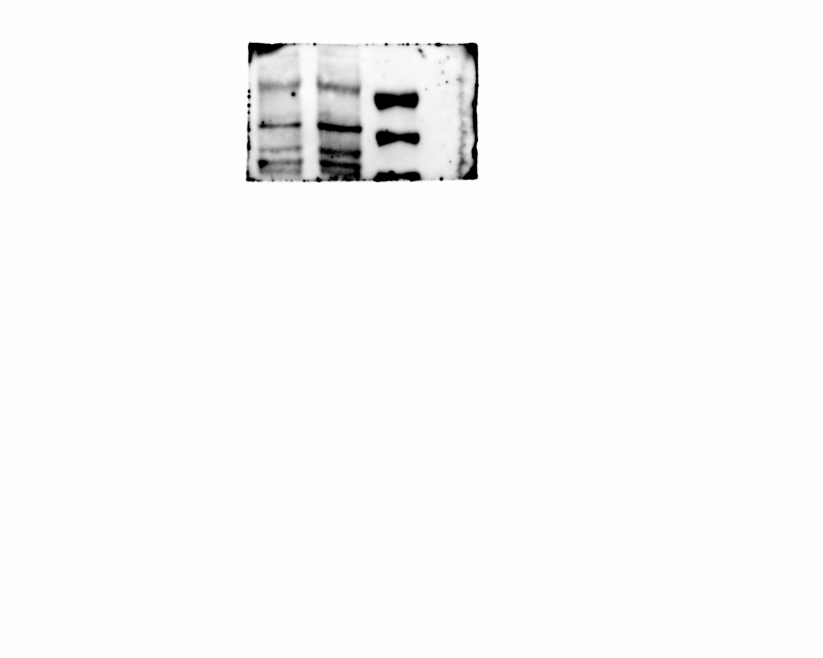


p-chk2


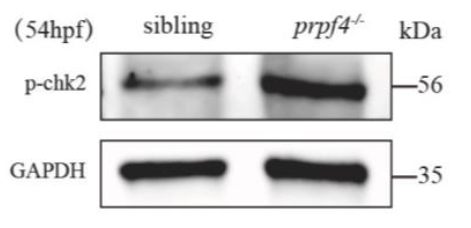


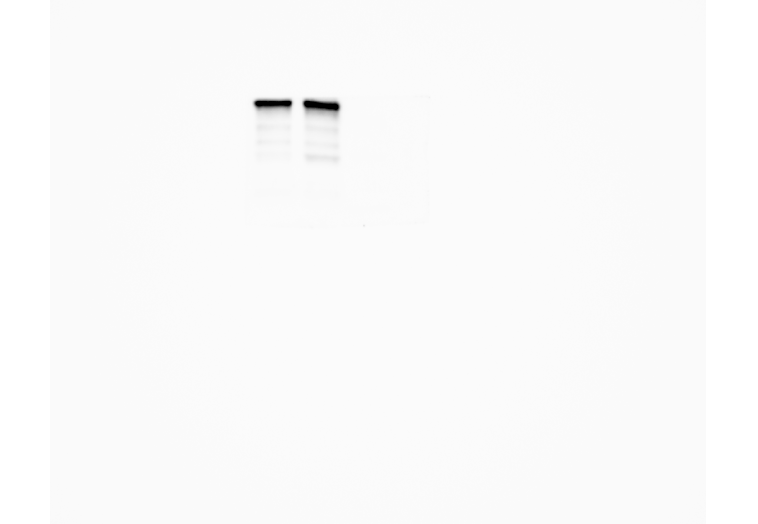


GAPDH


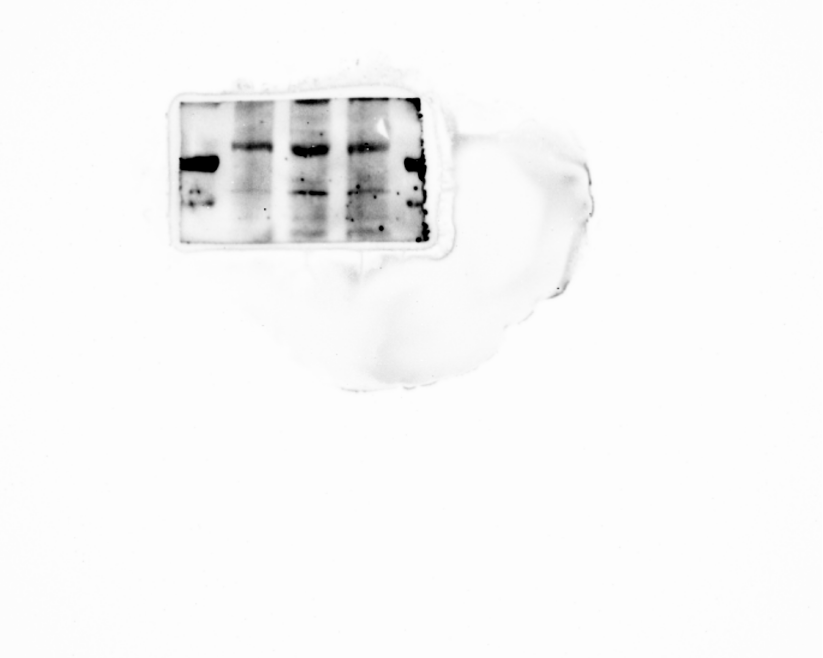


**Fig.4 D Gel supplementary**


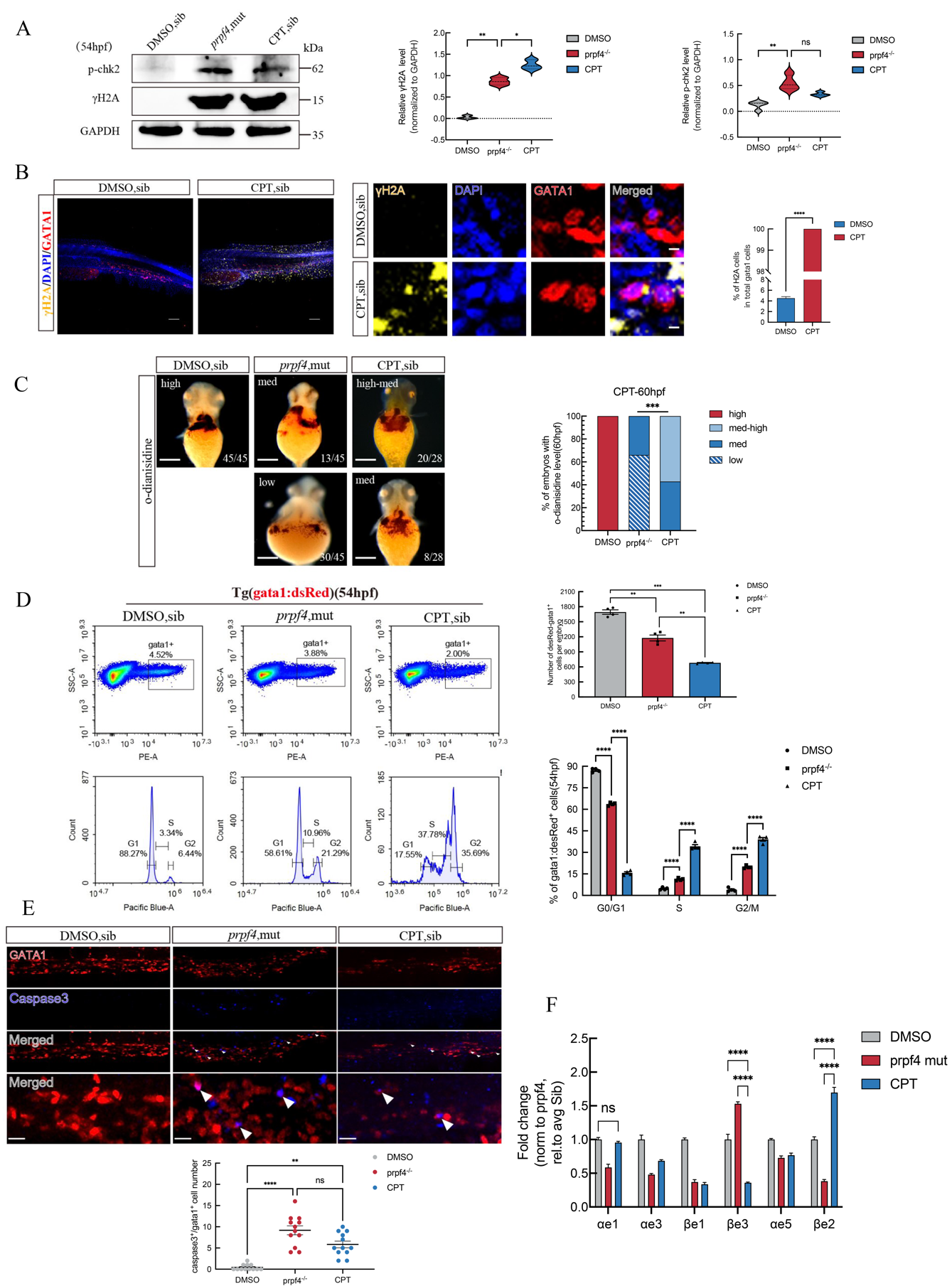


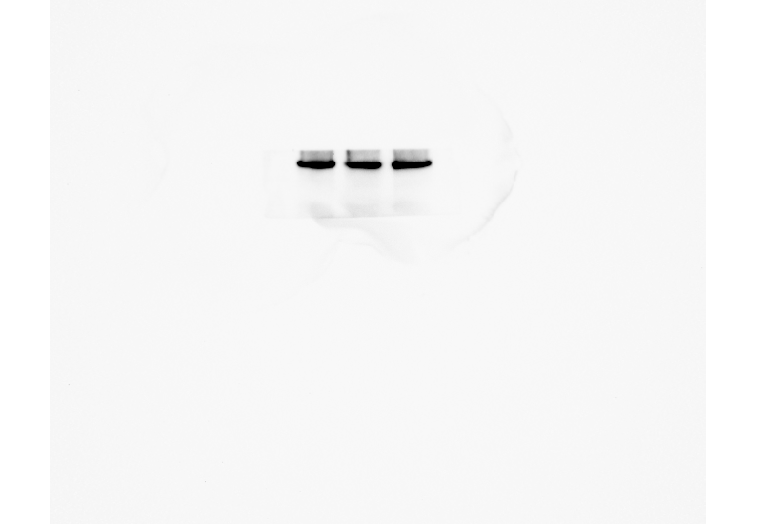

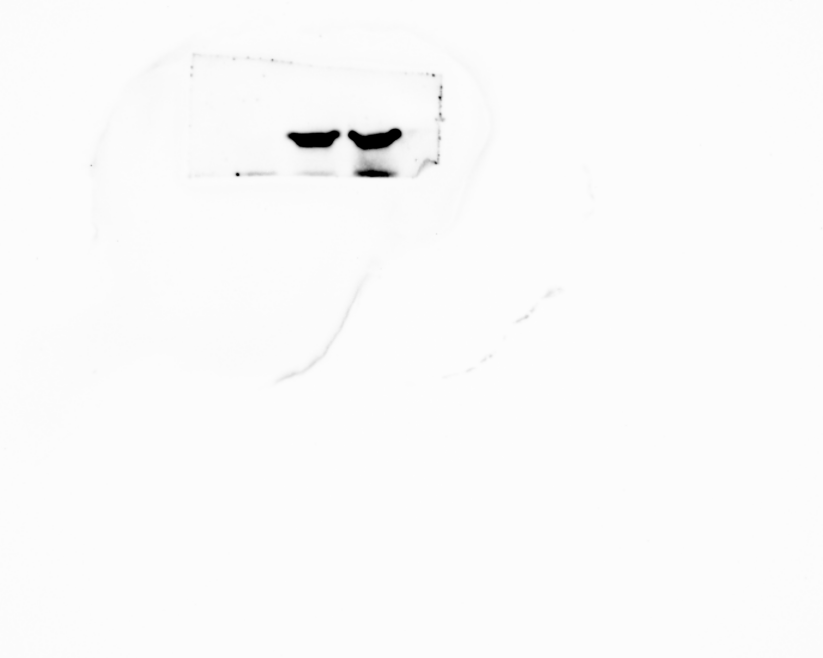


p-chk2

γH2A

GAPDH
